# New Monoterpenoid as the Sex Pheromone of Spanish Populations of the Longtailed Mealybug *Pseudococcus Longispinus* (Hemiptera: Pseudococcidae)

**DOI:** 10.1021/acs.jafc.4c00921

**Published:** 2024-05-21

**Authors:** Sandra Vacas, Ismael Navarro Fuertes, Víctor García-García, Javier Marzo Bargues, Antonio Abad-Somovilla, Jaime Primo, Vicente Navarro-Llopis

**Affiliations:** † CEQA-Instituto Agroforestal del Mediterráneo, Universitat Politècnica de València, Camino de Vera s/n, edificio 6C-5^a^ planta, Valencia, Valencia 46022, Spain; ‡ Universitat de València, Facultat de Químiques, Departamento de Química Orgánica, Dr. Moliner 50, Burjassot, Valencia 46100, Spain; § Ecología y Protección Agrícola SL, Pol. Ind. Ciutat de Carlet, Carlet, Valencia 46240, Spain

**Keywords:** semiochemicals, chemical ecology, insect attractant, integrated pest management

## Abstract

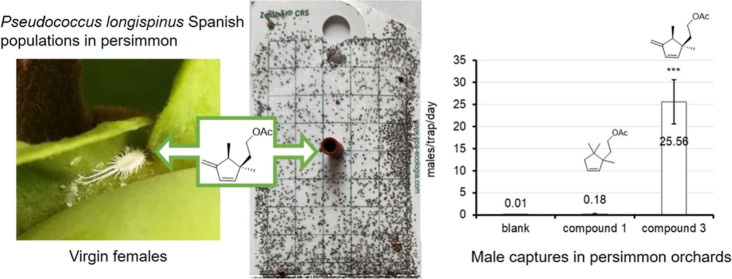

*Pseudococcus longispinus* (Targioni-Tozzetti) (Hemiptera: Coccoidea: Pseudococcidae), a polyphagous and cosmopolitan pest native to Australia, is a highly damaging pest for numerous crops of economic importance. The sex pheromone of this species (2-(1,5,5-trimethylcyclopent-2-en-1-yl)ethyl acetate), currently used for pest monitoring purposes, was not attractive to males in field experiments conducted in Spanish persimmon orchards infested with this mealybug. The virgin and mated female volatile profiles of these *P. longispinus* populations were studied by the volatile collection of effluvia in Porapak-Q. The resulting extracts were analyzed by gas chromatography coupled to mass spectrometry (GC–MS), revealing a new compound specific to virgin females and different from the previously described sex pheromone. Based on GC–MS data and nuclear magnetic resonance experiments, we envisaged monoterpene 2-(1,5-dimethyl-4-methylenecyclopent-2-en-1-yl)ethyl acetate as the new sex pheromone candidate, which was synthesized and shown to be attractive in the field to *P. longispinus* males of the Spanish population.

## Introduction

The long-tailed mealybug, *Pseudococcus longispinus* (Targioni-Tozzetti; Hemiptera: Coccoidea: Pseudococcidae), is a polyphagous and cosmopolitan pest that attacks a wide variety of plants (fruits, vegetables, and ornamental plants), causing serious damage to leaves, bark, branches, fruits, and roots.^1^ It has been described on 209 genera of 98 families of plants.^2^ Specifically, it is known as a very damaging pest for numerous crops of economic importance, among which are apple, pear, persimmon, grape, citrus, avocado, banana, and other tropical fruits.^3^ Although this species is native to Australia,^4,5^ it has spread throughout the world,^6^ reaching the Mediterranean region in the 19th century (1887 in Italy).^7^ It is currently present in 22 countries in continental Europe and the Mediterranean basin.^8^

As a mealybug, *P. longispinus* feeds on sap and produces honeydew that causes the proliferation of saprophytic fungi, as well as a decrease in the photosynthetic rate, loss of plant vigor, premature ripening, and fruit drop. In addition, due to its feeding habits, *P. longispinus* is capable of acquiring and transmitting viruses, being known for the effective transmission of some belonging to the families Betaflexiviridae^9^ and Closteroviridae,^10^ among others. In fact, the species *P. longispinus* is the only known vector of the grapevine leafroll-associated viruses GLRaV-5.^11^

The control of *P. longispinus* is currently based on the application of conventional chemical treatments, but their cryptic habits and the existence of overlapping generations make it difficult to manage. Moreover, since the ban by the European Commission on the use of methyl chlorpyrifos in January 2020, only a few active materials are currently available with limited efficacy, such as mineral oil and spirotetramat,^12^ the latter with an upcoming prohibition order in Europe. In this context, the lack of effective methods for controlling *P. longispinus* is evident, and farmers and producer associations need both direct control measures and tools for detection and population monitoring. These tools are of vital importance to improving mealybug control both in agricultural and ornamental ecosystems. However, monitoring usually consists of a laborious visual inspection of the plant material in search of the stages of the insect. Sticky traps baited with sex pheromones are a great tool for monitoring the flight of males in a more comfortable and sensitive way than visual inspection.^13^ Many of the economically important pseudococcid species reproduce sexually, with females producing a sex pheromone to attract conspecific males. So far, the chemical structure of the sex pheromones of 32 species of coccoid insects belonging to the families Diaspididae, Matsucoccidae, Margarodidae, and Pseudococcidae is known,^14^ and some of them are used for the detection and monitoring of their populations. The sex pheromone of the species *P. longispinus* was described by Millar et al.^15^ as the compound 2-(1,5,5-trimethylcyclopent-2-en-1-yl)ethyl acetate **1** and further tested in field trials in California, South America, New Zealand, Australia, and South Africa. However, preliminary tests carried out by our research group in persimmon orchards in Spain showed that traps baited with *P. longispinus* virgin females from populations collected in those orchards captured 100 times more males than traps baited with the synthetic pheromone already described (Supporting Information). According to this result, we decided to revise the sex pheromone blend composition of the Spanish populations of *P. longispinus* females collected from persimmon orchards to provide new compounds that allow the control and specific monitoring of this damaging mealybug.

## Materials and Methods

### Mealybug Stock Colony

The colony of *P. longispinus* was established in our facilities at the Universitat Politècnica de València (UPV, Valencia, Spain) using specimens collected from persimmon orchards in the Region of La Ribera (Valencia, Spain). Those specimens were identified as *P. longispinus* according to taxonomic keys and the features they showed.^16−18^ Several individuals are preserved in 70% ethanol in our facilities at UPV.

**Figure 1:**
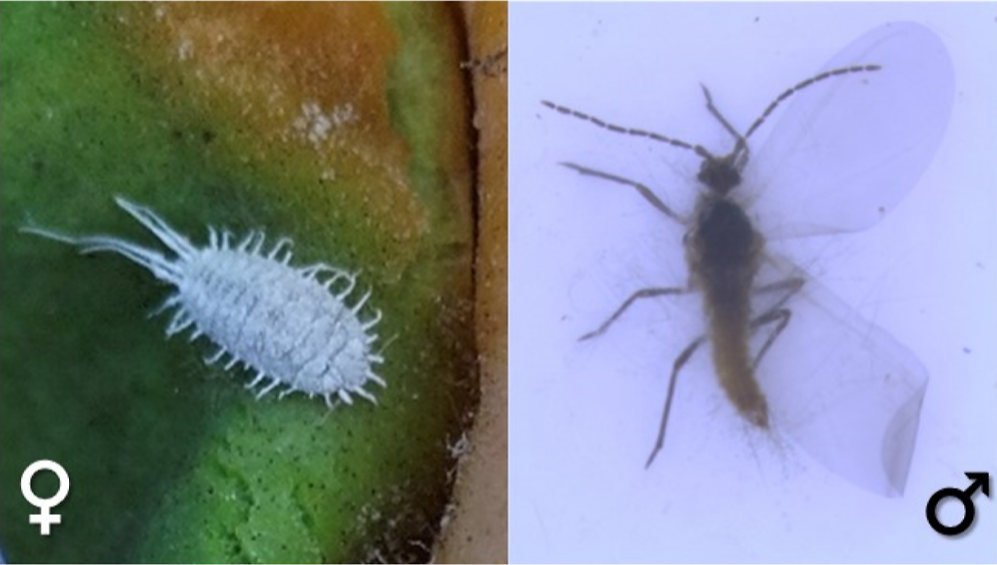
Pictures of female and male *Pseudococcus longispinus* collected in persimmon orchards.

Mealybugs were reared on pumpkins to establish the main stock colony. Insects were maintained in a rearing chamber under 14:10 (L/D) light-darkness conditions at 24 ± 2 °C with 40–60% relative humidity. Mealybugs for volatile collection were reared on organic green lemons, which were previously covered with paraffin wax around the midsection to delay their desiccation and prolong their useful life. Green lemons were preferred because of their size and the ease of detecting male pupae on their surface. Gravid females from pumpkins were gently transferred to green lemons. Then, newly hatched individuals established themselves on the surface of lemons and followed the developmental cycle. After the second instar stage, males produce a distinguishable cottony cocoon to pupate and transform into winged adults (Figure 1). Groups of lemons were visually inspected every 2–3 days for the presence of cocoons, which were manually removed with an entomological needle to leave lemons infested only with virgin females for volatile collection and profiling purposes. Other groups of lemons were left undisturbed to sample mated females. Lemons with virgin females were maintained in separated rooms under the same climate conditions.

### Collection of Volatiles

Groups of 5–6 lemons infested with approximately 300 *P. longispinus* females (virgin or mated separately) were placed in 5 L glass containers (25 cm high ×17.5 cm diameter flask), with a 10 cm open mouth and a ground glass flange to fit the cover with a clamp. The cover had a 29/32 neck on top to fit the head of a gas washing bottle to connect downstream a glass cartridge to trap effluents in 3 g of Porapak-Q (Supelco Inc., Torrance, CA, USA) adsorbent. Samples were collected continuously for 7–8 days (collection round) by using an ultrapurified-air stream, provided by an air compressor (Jun-air Intl. A/S, Norresundby, Denmark) coupled with an AZ 2020 air purifier system (Claind Srl, Lenno, Italy) to provide ultrapure air (amount of total hydrocarbons <0.1 ppm). In front of each glass container, an ELL-FLOW digital flowmeter (Bronkhorst High-Tech BV, Ruurlo, The Netherlands) was fitted to provide an air push flow of 300 mL/min during sampling. Trapped volatiles were eluted with 20 mL of pentane (Chromasolv, Sigma-Aldrich, Madrid, Spain), and the resulting extracts were concentrated to 500 μL under a gentle nitrogen stream prior to the chromatographic analysis. Lemons were replaced after each collection round (7–8 days), and ten rounds of virgin female collections were performed to obtain approximately 21,000 female-day equivalents (FDE), a sufficient quantity for the nuclear magnetic resonance (NMR) analysis.

All the resulting pentane extracts were analyzed by GC–MS in a Clarus 600 GC–MS (PerkinElmer Inc., Waltham, MA), equipped with a ZB-5MS fused silica capillary column (30 m × 0.25 mm i.d. × 0.25 μm; Phenomenex Inc., Torrance, CA). The oven was held at 40 °C for 2 min and then programmed at 5 °C/min to 180 °C before being raised to 280 °C at 10 °C/min and maintained at 280 °C for 1 min. Helium was used as a carrier gas with a flow of 1 mL/min. Detection was performed in the electron impact (EI) mode (70 eV) with the ionization source set at 200 °C. Spectrum acquisition was carried out in full scan mode [mass range *m*/*z* 35–500 atomic mass units (amu)], and chromatograms and spectra were recorded by means of GC–MS TurboMass software v. 5.4 (PerkinElmer Inc., Waltham, MA).

### Isolation of the Candidate Compound

After comparing the GC–MS volatile profiles of the virgin and mated samples (lemons infested with virgin and mated females, respectively), the virgin-specific compound was isolated by fractionation of the Porapak-Q pentane extracts by gravity column (300 × 15 mm i.d.) using the whole sample of ca. 21,000 FDE in 1 mL pentane. This sample was loaded into the column, and fractionation was performed using 12.5 g of silica gel as the stationary phase (40–60 μm) and eluents of pentane/diethyl ether mixtures (100:0, 95:5, 80:20, 50:50, and 0:100; 20 mL each). Forty fractions were collected, and they were all analyzed by GC–MS using the methods described in the previous section to identify those that exclusively contained the candidate compound. These fractions were gathered to collect the required quantity for the NMR analysis (ca. 60 μg). Then, the ^1^H NMR spectrum of the natural isolated compound was recorded by a Bruker 600 Ultrashield Plus spectrometer (Bruker, Billerica, MA) at a frequency of 600 MHz using C_6_D_6_ as the solvent with tetramethylsilane (TMS) as the internal standard.

### Synthesis of 2-(1,5,5-Trimethylcyclopent-2-en-1-yl)ethyl Acetate (1)

The sex pheromone compound previously described by Millar et al.^15^ was synthesized, according to the methods reported by Zou and Millar,^19^ to have enough quantity for field testing.

### Microreaction: Hydrolysis of the Natural 2-(1,5-Dimethyl-4-methylenecyclopent-2-en-1-yl)ethyl Acetate (3)

The pentane extract of a volatile collection sample of ca. 200 FDE (∼500 ng of pheromone) was hydrolyzed following related procedures.^20^ The extract was dried under a gentle nitrogen stream in a 2 mL GC glass vial. The residue was treated with a 0.5 M solution of KOH in methanol (150 μL) and stirred for 45 min at room temperature. After this time, water (0.5 mL) was added, and the solution was extracted with dichloromethane (0.5 mL twice). The combined organic phases were concentrated under nitrogen to ca. 100 μL volume before being submitted to GC–MS analysis.

### Synthesis of 2-(1,5-Dimethyl-4-methylenecyclopent-2-en-1-yl)ethyl Acetate (3)

#### General Procedures

The ^1^H and ^13^C spectra were recorded on a Bruker AC-300 spectrometer (Bruker, Billerica, MA) using CDCl_3_ or C_6_D_6_ as the solvent and TMS as the internal standard. Chemical shift values in ^1^H and ^13^C NMR are reported at δ (ppm) relative to chloroform (7.26/77.0 ppm) or benzene (7.16/128.4 ppm). High-resolution mass spectra (ESI-HRMS) were measured on a Waters Xevo Q-TOF spectrometer (Waters Corp., Milford, MA) coupled with an Acquity UPLC-PDA system (Waters Corp., Milford, MA) using ionization by electrospray (ESI). The ESI source operated in the positive ionization mode using leucine-enkephalin as the reference mass ([M + H] ^+^ ion *m*/*z* 556.2771). The sample (2 μL) was injected into a Waters Acquity BEH column (50 × 2.1 mm i.d., 1.7 μm) using MeOH as an isocratic eluent. The GC–MS analyses were performed with the aforementioned equipment (apparatus and column) and the following oven temperature program: 55 °C for 3 min, raised at 15 °C/min up to 180 °C and then at 35 °C/min up to 280 °C, held for 6 min. A helium flow of 1 mL/min and an injection volume of 1 μL were employed. Detection and spectral acquisition were performed as indicated above.

All reagents and solvents (reagent grade) were purchased from Sigma-Aldrich (Madrid, Spain) and employed with no further purification unless otherwise stated. In the case of reactions requiring anhydrous conditions, the solvents used were dried with the appropriate drying agents and distilled before use. Unless otherwise stated, all of the reactions sensitive to moisture and/or air were carried out under a nitrogen atmosphere. The solvent extracts of the reaction mixtures were dried over anhydrous MgSO_4_ or Na_2_SO_4_ and concentrated by rotary evaporation under reduced pressure. Crude products were purified by flash column chromatography using silica gel Merck 9385 (230–400 mesh). Thin-layer chromatography (TLC) was performed using Macherey-Nagel silica gel 60 F254 plates with a fluorescent indicator and UV light of 254 nm wavelength as the visualizing agent. Ceric ammonium molybdate and *p*-anisaldehyde were used as stains.

#### 5-(Hydroxymethyl)-3,4-dimethylcyclopent-2-en-1-one (**5**)

Ketone **4** (10 g, 0.091 mol), prepared according to Conia,^21^ was added dropwise to a solution of lithium isopropyl amide (LDA; 120 mL, 0.8 M) in THF at −30 °C, over 90 min. The solution was warmed up to 0 °C, and *p*-formaldehyde (8.17 g, 3 equiv) was added to the solution in one portion. After 30 min, the solution was poured into saturated ammonium chloride solution (90 mL) and extracted three times with EtOAc (50 mL). The combined organic phases were subsequently washed with solutions of 1 M HCl (20 mL × 2), saturated aqueous NaHCO_3_ (20 mL × 2), and brine and dried over anhydrous MgSO_4_. After solvent evaporation, the crude material was purified by column chromatography (Hexane-EtOAc 8:2) to afford alcohol **5** (5.09 g, 40% yield). ^1^H NMR (300 MHz, CDCl_3_) (Figure S1) d 5.89 (1H, m), 3.92–3.72 (2H, m), 2.65 (2H, m), 2.19 (1H, m), 1.70 (3H, m), 1.05 (3H, d, *J* = 7.2 Hz); ^13^C NMR (75 MHz, CDCl_3_) (Figure S2) δ 210.70, 182.91, 129.72, 62.01, 56.61, 42.14, 17.54, 17.37. MS (70 eV) *m*/*z*: 53 (3), 67 (2), 81 (8), 94 (7), 95 (7), 107 (1), 109 (7), 110 (4), 122 (4), 125 (2), 140 (7, M^+^). HRMS [ESI-TOF] calcd for C_8_H_12_O_2_ [M + H] ^+^ 141.0910, found 141.0909.

#### 3,4-Dimethyl-5-(((tetrahydro-2H-pyran-2-yl)oxy)methyl)cyclopent-2-en-1-one (**6**)

Alcohol **5** (5.09 g, 0.036 mol) was dissolved in anhydrous CH_2_Cl_2_ (60 mL) and 3,4-dihydropyran (DHP) (3.94 mL, 0.044 mol), and a catalytic amount of pyridinium *p-*toluenesulfonate (0.05 equiv) was subsequently added to the solution. The reaction mixture was warmed up to room temperature and monitored by TLC; after 23 h of continuous stirring, the solution was subsequently washed with 1 M NaHCO_3_ (20 mL × 2) and brine and dried over anhydrous MgSO_4_. After solvent evaporation, the crude mixture was purified by column chromatography (hexane-EtOAc 9:1) to afford the tetrahydropyranyl ether **6** (5.29 g, 65% yield). This compound and all those that follow it in the synthetic sequence that contain the tetrahydropyranyl moiety are diastereomeric mixtures, so some of the signals in the ^1^H NMR spectrum appear separated for each diastereomer. In these cases, for simplicity, only those signals attributable to one of the diastereomers are given. ^1^H NMR (300 MHz, CDCl_3_) (Figure S3) δ 5.88 (m, 1H), 4.63–4.55 (m, 1H), 3.89 (dd, *J* = 9.6, 7.2 Hz, 1H), 3.80 (m, 1H), 3.63 (dd, *J* = 9.7, 4.9, 1H), 3.55–3.43 (m, 1H), 2.90–2.72 (m, 1H), 2.21–2.11 (m, 1H), 2.09 (m, 3H), 1.79–1.39 (m, 6H), 1.22 (d, *J* = 3.1 Hz, 3H). ^13^C NMR (75 MHz, CDCl_3_) (Figure S4) δ 208.6, 181.92, 129.4, 99.70, 66.40, 62.77, 55.72, 48.76, 30.61, 25.53, 19.82, 17.85, 17.29. HRMS [ESI-TOF] calcd for C_13_H_20_O_3_ [M + Na] ^+^ 247.1305, found 247.1302.

#### 3,4-Dimethyl-5-(((tetrahydro-2H-pyran-2-yl)oxy)methyl)cyclopent-2-en-1-ol (**7**)

Compound **6** (5.29 g, 0.024 mol) was dissolved in anhydrous Et_2_O (10 mL) and dropwise added to a −20 °C cooled suspension of LiAlH_4_ (2.69 g, 3 equiv) in the same Et_2_O (65 mL) under an inert atmosphere (N_2_). The suspension was kept at this temperature while stirring for an additional 1.5 h. After this time, the solution was cooled to 0 °C, and Na_2_SO_4_·10H_2_O was added to the solution until gas evolution ceased. The mixture was filtered over Celite, and the solvent was evaporated. The residue obtained was chromatographed on silica gel (hexane-EtOAc 8:2) to afford compound **7** (5.15 g, 95% yield) as a 90:10 mixture of epimeric alcohols, as determined by NMR. ^1^H NMR (300 MHz, CDCl_3_) (Figure S5) δ 5.39 (m, 1H), 4.62 (m, 1H), 4.57 (bs, 1H), 3.89 (m, 1H), 3.81 (dd, *J* = 9.5, 8.5 Hz, 1H), 3.59–3.47 (m, 1H), 3.45 (dd, *J* = 9.6, 8.2 Hz, 1H), 2.04–2.01 (m, 1H), 1.95–1.71 (m, 3H), 1.69 (m, *J* = 1.5 Hz, 3H), 1.64–1.49 (m, 4H), 1.13 (d, *J* = 3.3 Hz, 3H). ^13^C NMR (126 MHz, CDCl_3_) (Figure S6) δ 147.09, 126.53, 99.16, 79.88, 69.49, 62.42, 57.61, 44.62, 30.89, 25.54, 19.84, 19.01, 14.82. HRMS [ESI-TOF] calcd for C_13_H_22_O_3_ [M + Na] ^+^ 249.1461, found 249.1458.

#### 3,4-Dimethyl-5-(((tetrahydro-2H-pyran-2-yl)oxy)methyl)cyclopent-2-en-1-yl Acetate (**8**)

Compound **7** (5.15 g, 0.023 mol) was dissolved in anhydrous CH_2_Cl_2_ (50 mL), and DMAP (0.05 equiv), Et_3_N (2.5 equiv), and acetic anhydride (0.029 mol, 2.87 mL) were added at room temperature. After 15 h of stirring, a saturated aqueous solution of NH_4_Cl (50 mL) was slowly added, the mixture was poured in EtOAc (40 mL), and the water phase separated. The aqueous phase was extracted with EtOAc, and the combined organic layers were successively washed with 1 M HCl (10 mL × 2), aqueous saturated NaHCO_3_ (10 mL × 2), and brine and dried over anhydrous MgSO_4_. After solvent evaporation, the residue was chromatographed on silica gel (hexane-EtOAc 9:1) to yield acetate **8** as a pale yellow oil (5.25 g, 70% yield). ^1^H NMR (300 MHz, CDCl_3_) (Figure S7) δ 5.42 (m, 1H), 5.30 (m, 1H), 4.55 (m, 1H), 3.86–3.66 (m, 2H), 3.53–3.27 (m, 2H), 2.32–2.18 (m, 1H), 1.96 (s, 3H), 2.02–1.98 (1H, m), 1.79–1.68 (m, 1H), 1.66 (m, 3H), 1.57–1.39 (m, 5H), 1.09 (d, *J* = 4.0 Hz, 3H). ^13^C NMR (75 MHz, CDCl_3_) (Figure S8) δ 171.11, 150.54, 122.45, 98.85, 81.56, 67.91, 62.05, 53.01, 44.57, 30.59, 25.50, 21.42, 19.38, 19.12, 14.89. MS (70 eV) *m*/*z* 51 (40), 43 (55), 55 (20), 57 (22), 60 (10), 65 (10), 67 (25), 77 (20), 79 (20), 85 (100), 91 (40), 93 (25), 95 (20), 106 (28), 107 (28), 109 (25), 124 (10), 141 (10), 183 (2), 208 (1), 226 (1). HRMS [ESI-TOF] calcd for C_15_H_24_O_4_ [M + Na] ^+^ 291.1567, found 291.1561.

#### 1,5-Dimethyl-4-(((tetrahydro-2H-pyran-2-yl)oxy)methyl)cyclopent-2-en-1-yl)acetic Acid (**9**)

A solution of acetate **8** (5.25 g, 0.016 mol) in dry THF (10 mL) was added dropwise to a LDA solution (0.5 M, 31.5 mL) in THF at −78 °C, over 90 min. The solution was warmed up to −50 °C, and chloro-*tert*-butyldimethylsilane (3.13 g, 0.021 mol) in dry THF (6 mL) was added to the solution in one portion. The resulting mixture was warmed up to room temperature during 3 h and then refluxed for 24 h. After cooling down to room temperature, MeOH (5 mL) was added to the solution and then acidified to pH 4–5 with an aqueous citric acid solution (20% w/v) and extracted three times with EtOAc (35 mL). The combined organic layers were subsequently washed with 1 M HCl (10 mL × 2), 1 M NaHCO_3_ (10 mL × 2), and brine and dried over anhydrous MgSO_4_. After solvent evaporation, the crude residue obtained was purified by chromatography (hexane-EtOAc 8:2) to give acid **9** as an oil (2.36 g, 55% yield). ^1^H NMR (400 MHz, CDCl_3_) (Figure S9) δ 9.96 (bs, 1H), 5.86 (dd, *J* = 5.8, 2.4 Hz, 1H), 5.68 (dd, *J* = 5.8, 1.6 Hz, 1H), 4.64–4.60 (m, 1H), 3.90–3.79 (m, 1H), 3.65 (dd, *J* = 9.4, 7.3 Hz, 1H), 3.56–3.46 (m, 1H), 3.31 (dd, *J* = 9.5, 6.9 Hz, 1H), 2.63–2.49 (m, 1H), 2.31–2.18 AB system (m, 2H), 1.86–1.75 (m, 1H), 1.74–1.44 (m, 6H), 1.20 (s, 3H), 1.01 (m, 3H). ^13^C NMR (101 MHz, CDCl_3_) (Figure S10) δ 178.95, 139.81, 131.52, 99.18, 70.88, 62.24, 51.87, 48.90, 48.57, 40.80, 30.73, 25.58, 25.35, 19.56, 12.65. MS (70 eV) *m*/*z* 55 (3), 67 (3), 79 (2), 85 (36), 91 (3), 107 (9), 121 (3), 166 (4), 238 (0.1, M^+^). HRMS [ESI-TOF] calcd for C_15_H_24_O_4_ [M + Na] ^+^ 291.1567, found 291.1566.

#### 2-(1,5-Dimethyl-4-(((tetrahydro-2H-pyran-2-yl)oxy)methyl)cyclopent-2-en-1-yl)ethan-1-ol (**10**)

Compound **9** (2.36 g, 8.8 mmol) was dissolved in anhydrous Et_2_O (10 mL) and dropwise added to a −20 °C cooled suspension of LiAlH_4_ (1 g, 3 equiv) in Et_2_O (35 mL) under an inert atmosphere (N_2_). The suspension was kept at this temperature while stirring for an additional 1.5 h. After this time, the solution was cooled to 0 °C, and Na_2_SO_4_·10H_2_O was added to the solution until hydrogen formation was no longer observed. The mixture was filtered over Celite, and the solvent was evaporated. The residue obtained was purified by chromatography (hexane-EtOAc 8:2) to afford alcohol **10** (1.66 g, 95% yield). ^1^H NMR (300 MHz, CDCl_3_) (Figure S11) δ 5.77–5.64 (m, 2H), 4.61 (t, *J* = 3.4 Hz, 1H), 3.93–3.78 (m, 2H), 3.71–3.64 (m, 2H) 3.63 (dd, *J* = 9.4, 7.7 Hz, 2H), 2.65–2.47 (m, 1H), 1.95–1.44 (m, 9H), 1.30 (bs, 1H), 1.06 (s, 3H), 1.01 (d, *J* = 7.1 Hz, 3H). ^13^C NMR (75 MHz, CDCl_3_) (Figure S12) δ 140.25, 131.42, 98.64, 71.18, 62.03, 60.55, 52.21, 48.66, 48.44, 39.45, 30.67, 26.12, 25.50, 19.58, 12.29. HRMS [ESI-TOF] calcd for C_15_H_26_O_3_ [M + H] ^+^ 255.1955, found 255.1949.

#### 2-(1,5-Dimethyl-4-(((tetrahydro-2H-pyran-2-yl)oxy)methyl)cyclopent-2-en-1-yl)ethyl Acetate (**11**)

Compound **10** (1.66 g, 6.5 mmol) was dissolved in anhydrous CH_2_Cl_2_ (30 mL), and DMAP (0.05 equiv), Et_3_N (2.5 equiv), and acetic anhydride (8.5 mmol, 0.82 mL) were added at room temperature. After 4 h of stirring, a saturated solution of ammonium chloride (20 mL) was slowly added. The mixture was poured into EtOAc (20 mL), and the water phase separated. The aqueous phase was extracted with EtOAc, and the organic layers were subsequently washed with 1 M HCl (5 mL × 2), 1 M NaHCO_3_ (5 mL × 2), and brine and dried over anhydrous MgSO_4_. After solvent evaporation, the crude product was purified by chromatography (hexane-EtOAc 9:1) to yield acetate **11** as a pale yellow oil (1.27 g, 66% yield). ^1^H NMR (300 MHz, CDCl_3_) (Figure S17) δ 5.75–5.59 (m, 2H), 4.60 (m,1H), 4.12–4.03 (m, 2H), 3.91–3.81 (m, 1H), 3.62 (dd, *J* = 9.4, 7.6 Hz, 1H), 3.54–3.46 (m, 1H), 3.27 (dd, *J* = 9.4, 7.2 Hz,1H), 2.61–2.48 (m, 1H), 2.02 (s, 3H), 1.89–1.72 (m, 1H), 1.67–1.43 (m, 5H), 1.07 (s, 3H), 1.02 (dd, *J* = 5.2 Hz, 3H). ^13^C NMR (75 MHz, CDCl_3_) (Figure S18) δ 171.27, 139.75, 131.59, 99.27, 71.18, 62.53, 62.34, 52.23, 48.83, 48.51, 34.94, 30.78, 26.00, 25.64, 21.21, 19.67, 12.52. MS (70 eV) *m*/*z* 43 (6), 55 (3), 57 (3), 67 (2) 77(1), 85 (37), 91 (3), 93 (3), 107 (6), 119 (2), 121 (1), 134 (5), 194 (1), 266 (0.1). HRMS [ESI-TOF] calcd for C_17_H_28_O_4_ [M + H] ^+^ 297.2060, found 297.2059.

#### 2-(4-(Hydroxymethyl)-1,5-dimethylcyclopent-2-en-1-yl)ethyl Acetate (**12**)

Acetate **11** (1.27 g, 4.3 mmol) was dissolved in MeOH (15 mL), and a catalytic amount of PTSA (0.01 equiv) was added to the solution. After 4 h of stirring, the solution was poured into 25 mL of CH_2_Cl_2_, the aqueous phase was extracted with CH_2_Cl_2_, and the combined organic layers were subsequently washed with an aqueous solution of NaHCO_3_ (10 mL × 2) and brine and dried over anhydrous Na_2_SO_4_. After solvent evaporation, the crude material obtained was purified by chromatography (pentane-Et_2_O 8:2) to give compound **12** as a pale yellow oil (0.82 g, 90% yield). ^1^H NMR (300 MHz, CDCl_3_) (Figure S19) δ 5.72 (dd, *J* = 5.9, 2.3 Hz, 1H), 5.67 (dd, *J* = 5.9, 1.4 Hz, 1H), 4.22–3.98 (m, 2H), 3.77 (dd, *J* = 10.6, 4.6 Hz, 1H), 3.53 (dd, *J* = 10.7, 6.0 Hz, 1H), 2.48 (m, 1H), 2.03 (s, 3H),1.72–1.47 (m, 3H), 1.09 (s, 3H), 1.01 (d, *J* = 7.1 Hz, 3H). ^13^C NMR (75 MHz, CDCl_3_) (Figure S20) δ 171.31, 141.17, 130.68, 64.77, 62.46, 54.72, 48.76, 46.98, 34.96, 25.86, 21.22, 12.25. HRMS [ESI-TOF] calcd for C_12_H_20_O_3_ [M + Na] ^+^ 235.1305, found 235.1304.

#### 2-(4-(Iodomethyl)-1,5-dimethylcyclopent-2-en-1-yl)ethyl Acetate (**13**)

Alcohol **12** (0.82 g, 3.86 mmol) was dissolved in anhydrous CH_2_Cl_2_ (15 mL), and PPh_3_ (1.41 g, 5.4 mmol), I_2_ (1.37 g, 5.4 mmol), and imidazole (0.86 g, 12.6 mmol) were subsequently added. After 5 h of stirring at room temperature, the resulting suspension was filtered off to remove the precipitate formed and subsequently washed with 1 M HCl (5 mL × 2), aqueous saturated NaHCO_3_ (5 mL × 2), and brine and dried over anhydrous Na_2_SO_4_. After solvent evaporation, the crude mixture obtained was purified by chromatography (pentane-Et_2_O 9:1) to give iodide **13** as a pale yellowish oil (0.88 g, 71% yield). ^1^H NMR (300 MHz, CDCl_3_) (Figure S21) δ 5.73 (dd, *J* = 5.8, 2.4 Hz, 1H), 5.62 (dd, *J* = 5.9, 1.6 Hz, 1H), 4.20–3.95 (m, 2H), 3.46 (dd, *J* = 9.2, 4.6 Hz, 1H), 3.07 (dd, *J* = 9.7, 7.3 Hz, 1H), 2.59–2.38 (m, 1H), 2.03 (s, 3H), 1.64–1.46 (m, 3H), 1.09 (s, 3H), 1.00 (d, *J* = 7.1 Hz, 3H). ^13^C NMR (75 MHz, CDCl_3_) (Figure S22) δ 171.26, 140.27, 133.11, 62.30, 53.54, 51.44, 49.67, 35.11, 25.82, 21.21, 12.31, 11.66. MS (70 eV) *m*/*z* 48 (8), 55(2), 67 (1), 79 (3), 91 (6), 93 (10), 108 (13), 119 (1), 135 (18), 235 (5), 247 (0.3), 262 (0.1). HRMS [ESI-TOF] calcd for C_12_H_19_IO_2_ [M + H] ^+^ 323.0503, found 323.0491.

#### 2-(−1,5-Dimethyl-4-methylenecyclopent-2-en-1-yl)ethyl Acetate (**3**)

Iodide **13** (0.88 g, 2.7 mmol) was dissolved in toluene (20 mL), 1,8-diazabicyclo[5.4.0]undec-7-ene (DBU; 4.9 mmol, 0.74 mL) was added, and the resulting solution was heated to 80 °C for 6 h. After this time, the solution was cooled down to room temperature and poured over Et_2_O (20 mL) and subsequently washed with aqueous citric acid solution 20% (w/v) (5 mL × 2), aqueous saturated NaHCO_3_ (5 mL × 2) and brine and dried over anhydrous MgSO_4_. After solvent evaporation, the crude mixture obtained was purified chromatography (pentane-Et_2_O 9:1) to give compound **3** as a pale yellowish oil (477 mg, 91% yield). ^1^H NMR (300 MHz, C_6_D_6_) (Figure S23) δ 6.03 (d, *J* = 5.7 Hz, 1H), 5.70 (dd, *J* = 5.7, 1.5 Hz, 1H), 4.88 (m 1H), 4.68 (m, 1H), 4.12–3.93 (m, 2H), 2.25 (qt, *J* = 7.2, 2.6 Hz, 1H), 1.67 (s, 3H), 1.53–1.41 (m, 2H), 0.95 (d, *J* = 7.2 Hz, 3H), 0.88 (s, 3H). ^13^C NMR (75 MHz, C_6_D_6_) (Figure S24) δ 170.03, 158.10, 145.25, 132.55, 102.88, 61.88, 48.92, 48.77, 36.12, 25.61, 20.62, 11.84. MS (70 eV) *m*/*z*: 43 (6), 53 (1), 65 (1), 79 (5), 91 (12), 107 (15), 119 (15), 134 (11), 194 (2, M^+^). HRMS [ESI-TOF] calcd for C_12_H_18_O_2_ [M + H] ^+^, 195.1380 found 195.1378.

#### 2-(1,5-Dimethyl-4-(((tetrahydro-2H-pyran-2-yl)oxy)methyl)cyclopent-2-en-1-yl)ethyl 4-Nitrobenzoate (**14**)

Compound **10** (0.1 g, 0.38 mmol) was dissolved in CH_2_Cl_2_ (2 mL), and DMAP (0.05 equiv), Et_3_N (2.5 equiv), and 4-nitrobenzoyl chloride (0.092 g, 0.5 mmol) were added at room temperature. The reaction mixture was poured into CH_2_Cl_2_ (15 mL) and subsequently washed with 1 M HCl (2 mL × 2), 1 M NaHCO_3_ (2 mL × 2), and brine and dried over anhydrous MgSO_4_. After solvent evaporation, the residue obtained was purified by chromatography (hexane-EtOAc 9:1) to yield compound **14** as a yellowish viscous oil (0.13 g, 90% yield). ^1^H NMR (300 MHz, CDCl_3_) (Figure S13) δ 8.28 (d, *J* = 8.8 Hz, 2H), 8.19 (d, *J* = 8.9 Hz, 2H), 5.81–5.62 (m, 2H), 4.61 (m, 1H), 4.50–4.30 (m, 2H), 3.87 (dd, *J* = 7.8, 3.8 Hz, 1H), 3.87–3.74 (m, 1H), 3.30 (dd, *J* = 9.4, 7.2 Hz, 1H), 2.65–2.54 (m, 1H), 1.94–1.43 (m, 10H), 1.14 (s, 3H), 1.08 (d, *J* = 5.1 Hz, 3H). ^13^C NMR (75 MHz, CDCl_3_) (Figure S14) δ 164.84, 150.61, 139.59, 135.96, 132.06, 130.80, 123.65, 98.79, 70.47, 64.04, 62.17, 52.30, 48.84, 48.56, 34.98, 30.80, 26.05, 25.62, 19.56, 12.45.

#### 2-(4-(Hydroxymethyl)-1,5-dimethylcyclopent-2-en-1-yl)ethyl 4-Nitrobenzoate (**15**)

Compound **14** (0.13 g, 0.32 mmol) was dissolved in MeOH (3 mL), and a catalytic amount of PTSA (0.01 equiv) was added to the solution. The reaction was stirred during 3 h, the solution was poured into 5 mL of CH_2_Cl_2_, and the aqueous phase was extracted with CH_2_Cl_2_. The combined organic layers were subsequently washed with 1 M NaHCO_3_ (2 mL × 2) and brine and dried over anhydrous Na_2_SO_4_. After solvent evaporation, the crude mixture obtained was purified by chromatography (pentane-Et_2_O 8:2) to give alcohol **15** as a pale white solid (0.08 g, 85% yield). ^1^H NMR (300 MHz, CDCl_3_) (Figure S15) δ 8.32–8.24 (m, 2H), 8.23–8.14 (m, 2H), 5.79 (dd, *J* = 5.9, 2.4 Hz, 1H), 5.73 (dd, *J* = 5.9, 1.4 Hz, 1H), 4.52–4.28 (m, 2H), 3.81 (dt, *J* = 9.6, 4.5 Hz, 1H), 3.57 (dt, *J* = 11.3, 6.1 Hz, 1H), 2.62–2.44 (m, 1H), 1.89–1.63 (m, 3H), 1.26 (dd, *J* = 6.4, 4.6 Hz, 1H) 1.16 (s, 3H), 1.06 (d, *J* = 7.1 Hz, 3H). ^13^C NMR (75 MHz, CDCl_3_) (Figure S16) δ 164.87, 140.99, 135.92, 131.02, 130.81, 123.69, 64.75, 63.98, 54.76, 48.85, 47.04, 35.05, 25.93, 12.31. HRMS [ESI-TOF] calcd for C_17_H_21_NO_5_ [M + H]^·+^ 320.1492, found 320.1483.

### Field Experiments

The response of *P. longispinus* males to 2-(1,5-dimethyl-4-methylenecyclopent-2-en-1-yl)ethyl acetate **3** was evaluated by means of a field trial carried out in a persimmon orchard located in the municipality of Alginet (Valencia, Spain). Substances were emitted from rubber septa (Ecología y Protección Agrícola SL, Carlet, Spain), which were loaded with 100 μg by impregnation with the corresponding hexane solutions of racemic 2-(1,5-dimethyl-4-methylenecyclopent-2-en-1-yl)ethyl acetate **3** or 2-(1,5,5-trimethylcyclopent-2-en-1-yl)ethyl acetate **1** (the pheromone compound described by Millar et al.^20^). The traps employed were 95 × 150 mm white sticky boards (Ecología y Protección Agrícola SL, Valencia, Spain). Six blocks of three traps were installed to test the above-mentioned substances, including a blank trap (baited with a rubber septa only loaded with hexane). Within each block, traps were hung at a height of 1.5 m and were spaced 10 m apart, with each block at least 30 m apart. The traps were revised fortnightly, and the number of captured males was counted under a stereomicroscope (Stemi 508; Zeiss, Oberkochen, Germany) at 50× magnification.

The number of captured males with each substance was compared using generalized linear mixed models (GLMM). For this purpose, the *glmer* function from the lme4 package was employed by assuming the poison error distribution. Models were constructed with the fortnightly captures as the dependent variables, substance and time (week of the study period) as fixed factors, and the block (experimental replicate) as the random factor. The significance of substance effects was assessed by removing them from each model and comparing models with likelihood ratio tests. The *glht* function in the multcomp package was then used to perform Tukey HSD tests for post hoc pairwise comparisons (*P* < 0.05).

## Results

### Chemical Analysis and Structure Elucidation

**Figure 2:**
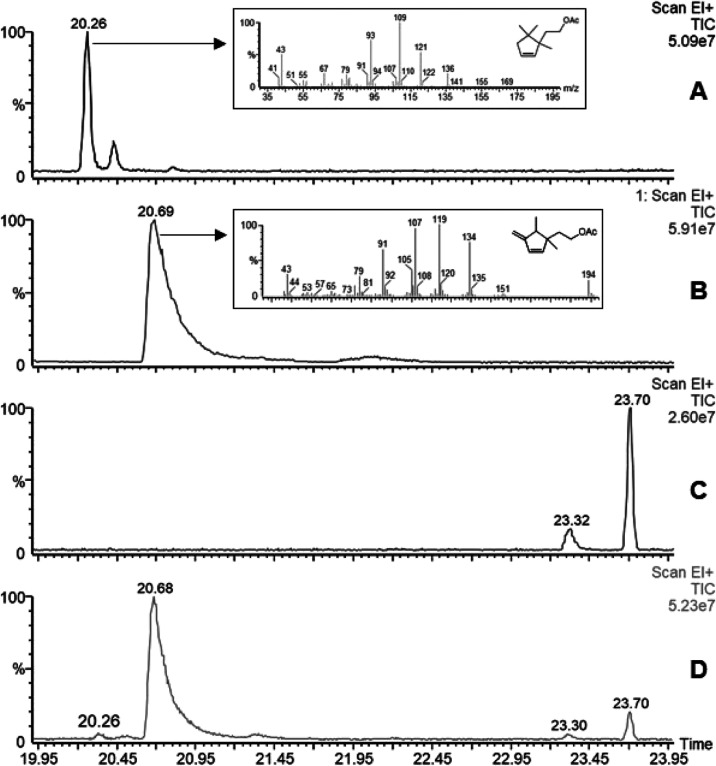
GC/MS chromatograms showing (A) synthetic sample of 2-(1,5,5-trimethylcyclopent-2-en-1-yl)ethyl acetate **1** at 20.26 min and (B) synthetic sample of 2-(1,5-dimethyl-4-methylenecyclopent-2-en-1-yl)ethyl acetate **3** at 20.68 min. None of these substances appeared in (C) mated female volatile collection but matched the peaks detected in (D) virgin female volatile collection.

The chromatographic volatile profiles of lemons infested with females, either virgin or mated, revealed an unknown, major, virgin-specific compound at 20.69 min, as well as another minor one at 20.26 min (1% regarding the area of the major compound) (Figure 2). The latter was identical to 2-(1,5,5-trimethylcyclopent-2-en-1-yl)ethyl acetate **1**, described by Millar et al.,^15^ in its GC retention time and MS spectra (Figure 2A). By sampling 21,000 FDE, ca. 60 μg of the major compound was collected, which suggests that a single *P. longispinus* female emitted approximately 2.86 ng on average of this substance. The yield of **1** was approximately 100 times lower. Neither of the two substances mentioned was found in the samples of mated females.

**Figure 3:**
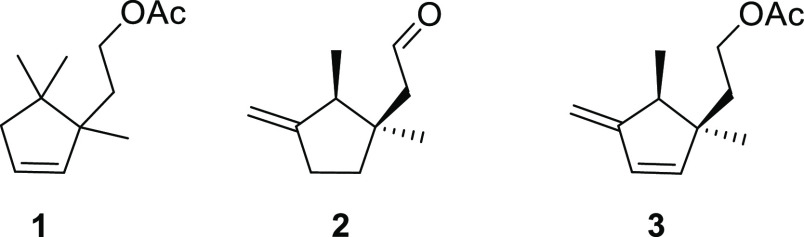
Structures of the sex pheromone of *P. longispinus* (**1**) described by Millar et al.,^15^*D. brevipes* (**2**),^22^ and Spanish populations of *P. longispinus* (**3**).

At first, our main hypothesis was to find a related structure to 2-(1,5,5-trimethylcyclopent-2-en-1-yl)ethyl acetate **1**, originally described by Millar et al.^15^ (Figure 3). Assuming the peak at *m*/*z* 194 to be the molecular ion (Figure 2B), the mass spectrum of this compound shows its base peak at *m*/*z* 119 and main fragments at *m*/*z* 134, 107, 91, and 43. The characteristic loss of 60 amu to give *m*/*z* 134, together with the presence of the ion at *m*/*z* 43, suggested the presence of an acetate ester. As expected from other sex pheromones of related species, the fragmentation pattern seemed to be related to a monoterpenoid structure. A micro saponification of a sample of the isolated compound was performed, giving a new compound with a molecular ion of *m*/*z* 152, and main fragments at *m*/*z* 137, 121, 119, 107, and 91 (Figure S25), confirming the presence of the acetate moiety. Interestingly, the mass loss fragments of this alcohol followed a very close fragmentation pattern when compared with the substituted cyclopentane alcohol obtained by reduction of the aldehyde pheromone **2** (Figure 3) of the pineapple mealybug *Dysmicoccus brevipes* (Cockerell),^22^ showing mass fragments at *m*/*z* 139, 121, 109, and 93. This fact suggested a structural similarity of both compounds, but with an extra unsaturation (four in total) in the case of the newly isolated compound, with a possible molecular formula of C_12_H_18_O_2_.

Analysis of the ^1^H NMR, ^13^C NMR, and multiplicity-edited HSQC spectra of the natural isolated compound (Figure S26) showed characteristic signals for an unsaturated monoterpene. In particular, two coupled protons were clearly defined at δ 6.02 and 5.70 (each d, *J* = 5.8 Hz), which correlate with olefinic CH carbons at δ 145.2 and 132.6 ppm, respectively, clearly indicating the presence of a *cis* olefinic moiety. On the other hand, the protons at δ 4.87 (d, *J* = 2.2 Hz) and 4.68 (bs) ppm showed a single correlation with a carbon signal in the alkene region (δ 102.9 ppm), indicative of the presence of an exo methylene moiety. The only additional signal in the most deshielded region of the proton spectrum is a multiplet centered at δ 4.03 ppm, correlated with the signal of a methylene carbon atom at about δ 62 ppm, which is compatible with a methylene group attached to an acetoxy moiety, whose methyl group is also observed in the proton and carbon NMR spectra at δ 1.67 and 20.6 ppm, respectively. The rest of signals of the proton spectrum appear in the resonance region of aliphatic protons, highlighting a multiplet centered at δ 2.25 ppm, correlated with the signal of a methyne carbon atom at about δ 49 ppm, compatible with the presence of an allylic CH group. The most shielded signals in the proton spectrum correspond to two methyl groups, one at δ 0.95 ppm (d, *J* = 7.2 Hz), correlated with the signal of a carbon atom at δ 11.8 ppm, compatible with a CH_3_ group attached to the methyne carbon atom, and another at δ 0.88 ppm (s), correlated with the carbon signal at δ 25.6 ppm, compatible with a CH_3_ group on a quaternary center.

The mass spectra and NMR data discussed above allowed us to tentatively suggest that the chemical structure of the new pheromone is based on a 3-methylene-cyclopentene framework bearing a methyl group at position C–4 and a methyl and a 2-acetoxyethyl groups at position C–5. We envisage a structure such as **3** [2-(1,5-dimethyl-4-methylenecyclopent-2-en-1-yl)ethyl acetate], initially assuming a *trans* relationship between the methyl groups at C–4 and C–5 as in the previously mentioned sex pheromone of the pineapple mealybug. A confirmatory synthesis of the proposed structure was carried out in order to confirm the identity of this new sex pheromone.

### Synthesis and Identification of the Pheromone

**Figure 4:**
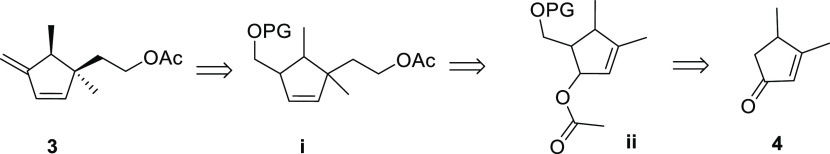
Retrosynthesis of compound **3** from cyclopentenone **4**.

The synthetic strategy followed for the preparation of compound **3** is outlined in the retrosynthetic analysis depicted in Figure 4. In principle, compound **3** could be synthesized from an intermediate such as **i** using a base-mediated elimination reaction upon transformation of the protected hydroxyl group into a suitable leaving group. This intermediate could be synthesized from allylic acetate **ii** using a Claisen-Ireland [3,3]-sigmatropic rearrangement of the silyl enol ether derivative of the acetate moiety as key step, giving thus access to the quaternary carbon atom present in the target structure, a strategy previously used by Millar et al.^15^ for the synthesis of the related skeleton of the *P. longispinus* sex pheromone **1**. Acetate **ii** could be achieved from readily available cyclopentenone **4**(21) via hydroxymethylation, followed by appropriate protection of the introduced hydroxyl group, reduction of the carbonyl group, and acetylation of the resulting allylic alcohol.

**Figure 5:**
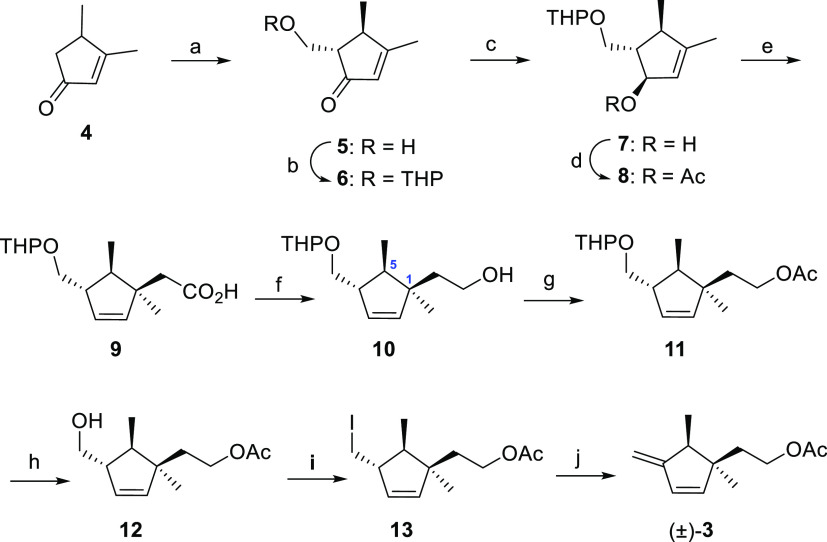
Synthesis of **3**: (a) LDA, THF, −30 °C, 90 min, then (CHO)_*n*_, 0 °C, 30 min, 40%; (b) DHP, PPTS, CH_2_Cl_2_, rt, 23 h, 65%; (c) LiAlH_4_, Et_2_O, −20 °C, 1.5 h, 95%; (d) Ac_2_O, Et_3_N, DMAP, CH_2_Cl_2_, rt, 15 h, 70%; (e) i. LDA −78 °C, 90 min, ii. TBDMSCl, THF, −50° to rt, 3 h, iii. Reflux, 24 h; iv. Citric acid, MeOH–H_2_O, 55%; (f) LiAlH_4_, THF, 0 °C, 2 h, 95%; (g) Ac_2_O, Et_3_N, DMAP, CH_2_Cl_2_, rt, 4 h, 66%; (h) PTSA, MeOH, rt, 4 h, 90%; (i) PPh_3_, I_2_, imidazole, CH_2_Cl_2_, rt, 5 h, 71%; (j) DBU, PhMe, 80 °C, 6 h, 91%.

The synthesis of **3** commences with the hydroxymethylation of cyclopentenone **4** (Figure 5) that was undertaken following the procedure previously described for related substrates.^23^ Thus, treatment of the enolate generated from the reaction of **4** with LDA with paraformaldehyde afforded a 95:5 racemic mixture of *anti*/*syn* diastereoisomeric 5-hydroxymethylated cyclopentenones, which could be separated by column chromatography to afford the pure *anti* diastereomer **5** in 40% yield. Protection of the hydroxyl group of **5** as tetrahydropyranyl ether under standard conditions with DHP and catalytic PPTS gave **6** in 65% yield. Regioselective reduction of enone **6** with LiALH_4_ in Et_2_O gave the secondary alcohol **7** as a 95:5 mixture of epimers at the carbinol center, as deduced from the analysis of the ^1^H NMR spectra (for clarity, only the major β-epimer is drawn in Figure 5). This high stereoselectivity is in concordance with the result observed in the reduction of structurally related cyclopentenones^24,25^ and could be probably rationalized in terms of the steric effect exerted by the methyl group at C–4 and the potential coordination of the reducing agent to the tetrahydropyranyl ether moiety. In our hands, no separation of this mixture of epimeric alcohols using conventional silica gel chromatography was possible. Conversion of **7** into its corresponding acetate afforded **8** in a 70% yield. Ireland-Claisen rearrangement of the trimethylsilyl enol ether derived from acetate **8**, followed by acid treatment of the reaction mixture, gave carboxylic acid **9** in a modest 55% yield, but that is in agreement with the results obtained in rearrangements of structurally related substrates.^19^ The *trans* relative disposition of the methyl groups in the rearranged product **9** was inferred from NOE experiments that showed that irradiation of both the AB system contiguous to the carboxylic group (δ 2.26 ppm), and the methyl group at the quaternary C–1 position (δ 1.21 ppm) caused a weak but significant increase in the intensity of the methyl group (δ 1.04 ppm) and the methyne proton (δ 1.67 ppm) at C–5, respectively. The determination of the relative stereochemistry of acid **9** also served to confirm the stereochemistry initially assigned for the major epimeric alcohol formed in the reduction reaction of enone **6** since the Ireland-Claisen rearrangement takes place with chirality transfer from the C–O to the C–C bond.

Once the carbon framework of compound **3** was completed, the rest of the steps to complete its synthesis involved only modifications of the functional groups present in **9**. The first functional transformation carried out was the reduction of the carboxylic group to the corresponding alcohol, a reduction that was carried out by treating acid **9** with LiAlH_4_ in Et_2_O to afford the primary alcohol **10** in a 95% yield which was acylated using acetic anhydride, Et_3_N, and DMAP under usual conditions to obtain the corresponding acetate **11** in 66% yield. Next, the tetrahydropyranyl group was removed by treatment of **11** with MeOH and catalytic PTSA to give alcohol **12** in 90% yield, whose hydroxyl group was replaced by an iodine atom using the iodine–triphenylphosphine–imidazole reagent. Finally, iodide-obtained **13** was subjected to a bimolecular elimination process to generate the exocyclic double bond using DBU as a base and relatively mild temperature conditions. The E2 reaction proceeded quite efficiently to provide the racemic form of cyclopentadiene **3** in 91% yield.

**Figure 6:**
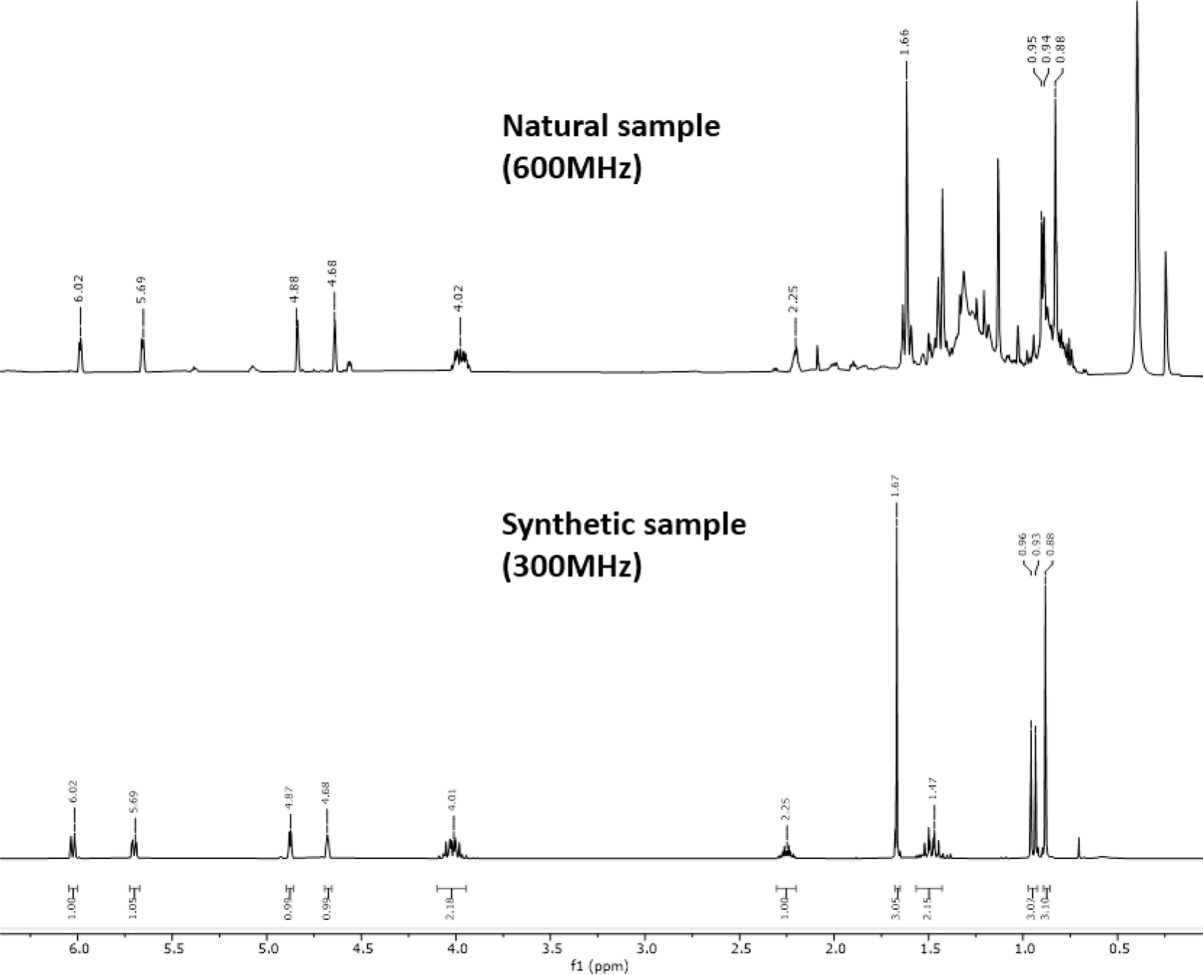
^1^H NMR spectra of the natural and synthetic compound **3** samples were fully coincident.

The ^1^H NMR spectroscopic data of synthetic diene **3** in C_6_D_6_ afforded a set of main signals, which were fully coincident with those observed for the natural isolated compound from virgin female volatile emissions (Figure 6). The identity of both compounds was further corroborated by GC and the mass fragmentation pattern which was virtually identical for the synthetic and natural samples.

**Figure 7:**
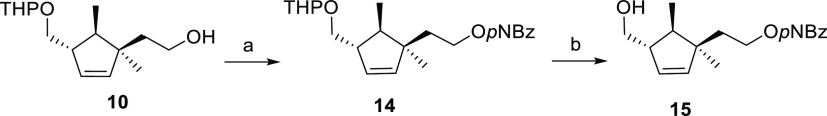
Crystalline derivative of alcohol **10** (a) ClCOC_6_H_4_-*p*-NO_2_, Et_3_N, DMAP, CH_2_Cl_2_, rt, 1 h, 90%. (b) PTSA, MeOH, rt, 3 h, 85%. THP: Tetrahydropyranyl group; *pNBz*: *p-nitrobenzoyl group* |COC_6_H_4_-*p*-NO_2_].

To unequivocally confirm the relative stereochemistry assigned to the generated stereogenic centers based on NMR data, we performed an X-ray diffraction analysis of a crystalline derivative of alcohol **10**. The derivative chosen was the corresponding *p*-nitrobenzoyl ester **15** (Figure 7) that was prepared in two steps from alcohol **10**. First, treatment of **10** with *p*-nitrobenzoyl chloride and Et_3_N to give ester **14** with a 90% yield, followed by the removal of the THP group with PTSA in MeOH to afford ester-alcohol **15** in 85% yield. An appropriate sample for single-crystal X-ray diffraction analysis was obtained when the solid *p*-nitrobenzoyl ester was crystallized from cold hexane, whose ORTEP diagram is shown in Figure S28, confirming the relative configuration initially proposed for all the stereogenic centers of the cyclopentene skeleton.

### Field Activity

**Figure 8:**
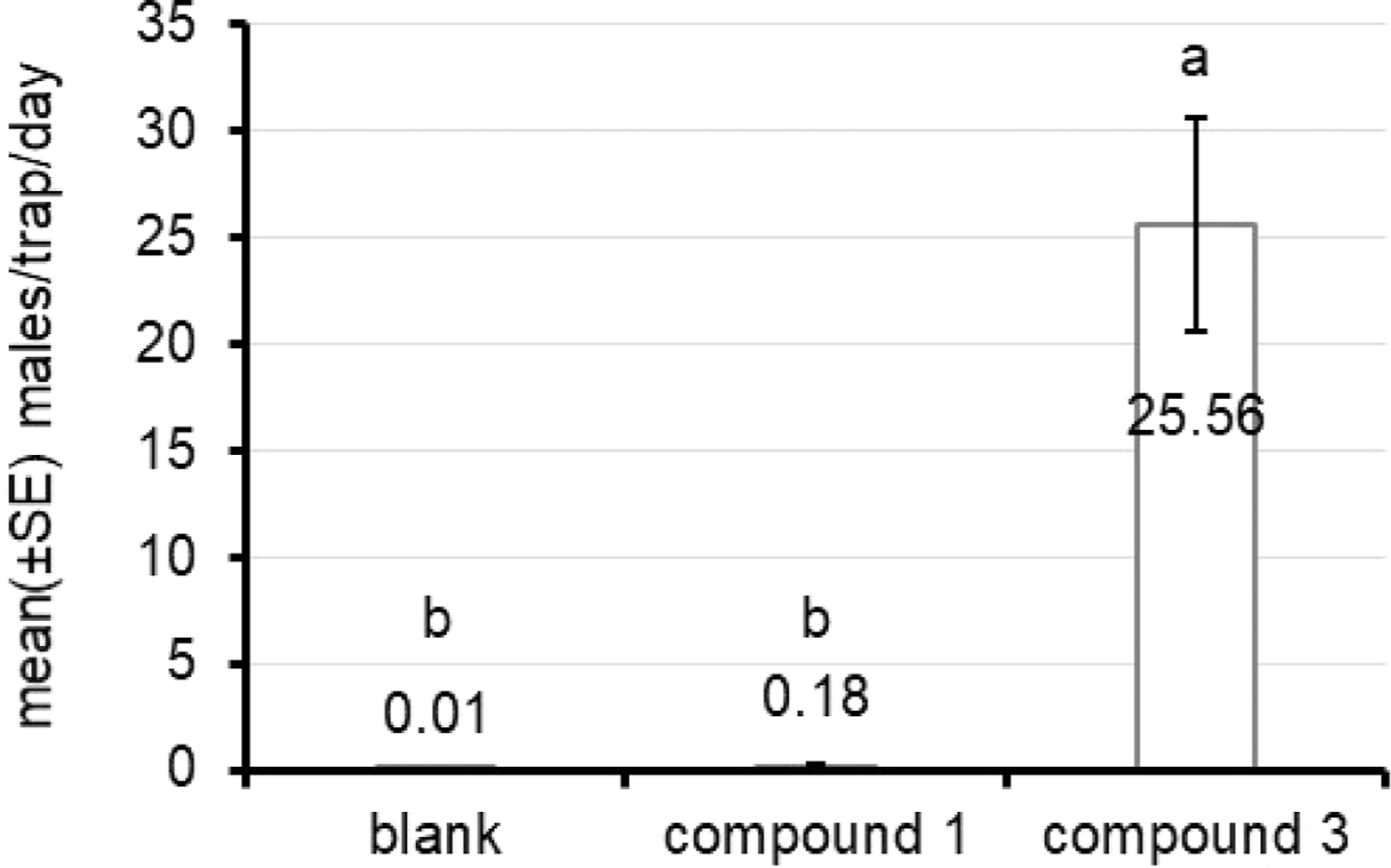
Mean (±SE) number of males captured per trap and day in blank traps (no bait) and those baited with 2-(1,5,5-trimethylcyclopent-2-en-1-yl)ethyl acetate (compound **1**) and 2-(1,5-dimethyl-4-methylenecyclopent-2-en-1-yl)ethyl acetate (compound **3**). (***) It is significantly different from the others according to GLMM (Tukey HSD test at *P* < 0.05).

In the field, blank traps and traps baited with compound **1** captured only 1 and 26 males, respectively, throughout the trial, whereas a total of 4178 males were captured in traps baited with compound **3** (Figure 8). This confirms the identity of the natural sex pheromone compound previously determined by the analytical procedures.

## Discussion

Compound **1** (2-(1,5,5-trimethylcyclopent-2-en-1-yl)ethyl acetate), originally identified as the sex pheromone of the long-tailed mealybug by Millar et al.,^15^ has been recently employed in field trials conducted in New Zealand,^26^ effectively capturing males and finding an economic optimal dose of 20 μg when employing the racemic mixture of **1** for monitoring *P. longispinus*. They also found that the non-natural enantiomer did not affect the attraction of the natural (S)-(+)-enantiomer detrimentally. The insects collected in persimmon orchards of Eastern Spain to establish our stock colony were taxonomically identified as belonging to *P. longispinus* species according to several keys.^16−18^ However, traps baited with the reported synthetic pheromone **1** captured only a few males in those persimmon orchards, whereas traps baited with virgin females of the local populations captured 100 times more males than compound **1**. This result strongly suggested that the sex pheromone blend of *P. longispinus* Spanish populations was different from that previously reported. Accordingly, our study on the volatile profile of *P. longispinus* females collected in Spanish persimmon orchards revealed a new monoterpenoid, 2-(−1,5-dimethyl-4-methylenecyclopent-2-en-1-yl)ethyl acetate (compound **3**), as the major sex pheromone component of these insect populations with a powerful attractant activity. Interestingly, a minor (ca. 1%) quantity of compound **1** was also detected in virgin female effluvia but demonstrated very poor field attraction by itself.

**Figure 9:**
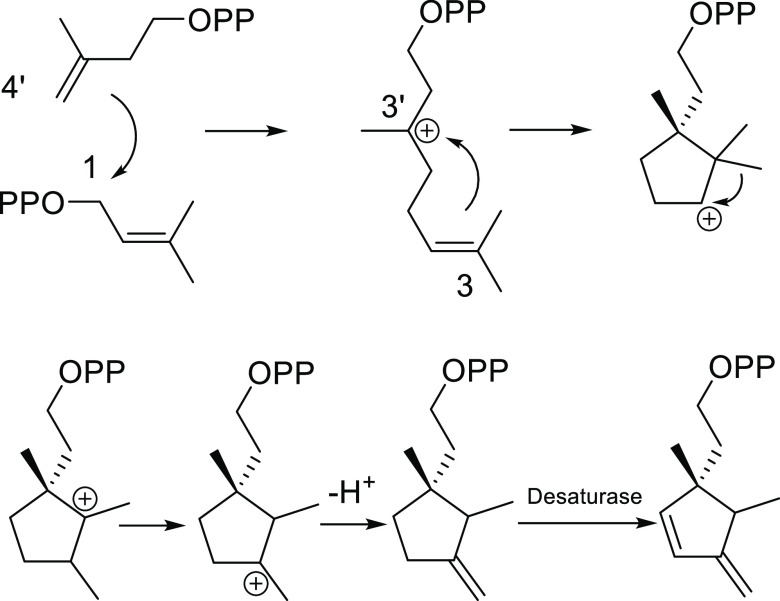
Suggested biosynthetic pathway for compound **3**.

Following the structural patterns observed in mealybug sex pheromones identified until now, compounds **1** and **3** belong to the monoterpenes. However, the described new compound possesses a 1,2,3 trimethyl cyclopentane skeleton, similar to the one described by Tabata et al.^22^ for the sex pheromone of *D. brevipes*. The biosynthetic origin of compound **3** could be rationalized from a regular head-to-tail 4′–3 linkages between the isoprene units, followed by a 3′-3 connection and Wagner–Meerwein rearrangements. The first insaturation could be explained via elimination reaction and, presumably, as in the case of the pheromone of *Delottococcus aberiae* (De Lotto),^20^ a desaturase is necessary to introduce second unsaturation into the cyclopentane framework (Figure 9).

It is well established that the already reported structural diversity of regular and irregular monoterpenoids of Coccoidea sex pheromones does not always follow the phylogenetic relationship between closely related mealybug species, such as in the case of *D. brevipes* and *D. neobrevipes* Beardsley, the former having a cyclopentane aldehyde and the last an acyclic acetate.^22^ Moreover, intraspecies pherotype blend modulation or divergence is not uncommon and has been also described for moths (*E/Z* isomeric ratio changes in *Ostrinia nubilalis* (Hübner)^27^) or mealybug species, such as *Planococcus ficus* (Signoret), whose Californian populations produce a single-component sex pheromone, while the Israeli ones produce an additional compound.^28^ Isolation of populations during more than a century may explain such phenotype changes. In the case of *P. longispinus*, Californian and European populations were reported in 1918^29^ and 1866,^7^ respectively, although Australia was concluded to be the native habitat of the species, on the basis of its low population density and high parasitism rates.^5^ Indeed, it is important to mention that other factors, such as the host plant, may influence the species-specific sex pheromone evolution, being more plastic than previously assumed.^30^ Considering that *P. longispinus* is a polyphagous species, this fact could support the existence of, at least, two different pherotypes pointing to a different cyclization process of the isoprene units in the sex pheromone production that is operating in each population. This fact should be studied by using molecular techniques, trying to identify the mutations that produce these pheromone blend modulations in the *P. longispinus* populations originating from Australia, America, and Europe.

Mealybug sex pheromones could be powerful tools for controlling pest populations, and some examples of their use have been recently described. In particular, the mating disruption technique has been developed and tested for *P. ficus*,^31^*Pseudococcus calceolariae* (Maskell),^32^ or *Planococcus kraunhiae* (Kuwana),^33^ but only for *P. ficus* there is a commercially available mating disruption treatment for controlling the pest.^34,35^ This is probably due to a more cost-effective synthetic pheromone according to their structures.^36^ Compound **1**, the first reported sex pheromone of *P. longispinus*, has been shown to be useful for population monitoring purposes;^37−39^ however, no direct control method based on this substance has been described yet, probably due to its complicated and expensive synthetic process.

The absolute configuration of the natural compound **3** is not yet established, but the racemic mixture has proven effective to capture *P. longispinus* males in our field experiments. The use of this new monoterpenoid to monitor pest populations can be immediately implemented, improving the sensitivity of the current visual inspection procedures. However, further studies are needed to evaluate the activity of the combination of compounds **3** and **1**, as detected in virgin female effluvia, as well as the potential of direct control techniques based on this new sex pheromone, such as mating disruption or attract and kill.

## Supporting Information Available

^1^H NMR and ^13^C NMR spectra of the synthetic compounds; GC–MS spectrum of 2-(1,5-dimethyl-4-cyclopent-2-en-1-yl)ethanol; multiplicity-edited HSQC spectra of the natural sex pheromone isolated from *P. longispinus* female Spanish populations; multiplicity-edited HSQC spectra of compound **3**; ORTEP diagram for compound **15**; mean (±SE) number of males captured per trap and week in traps baited with virgin females and those baited with 2-(1,5,5-trimethylcyclopent-2-en-1-yl)ethyl acetate **1**; and crystal data and structure refinement for compound **15** (PDF)

## References

[ref1] Raja S.; Gillani W.; Copland M. Effect of different temperatures and host plants on the biology of the long-tailed mealybug *Pseudococcus longispinus* (Targioni and Tozzetti) (Homoptera: Pseudococcidae). Biol. Sci. 2011, 54, 142–151. 10.52763/PJSIR.BIOL.SCI.54.3.2011.142.151.

[ref2] García-Morales M.; Denno B. D.; Miller D. R.; Miller G. L.; Ben-Dov Y.; Hardy N. B. ScaleNet: A literature-based model of scale insect biology and systematics. Database 2016, 2016, bav118 10.1093/database/bav118. 26861659 PMC4747323

[ref3] Mani M.; Shivaraju C. Management of Mealybugs in Agricultural and Horticultural Crops. In Mealybugs and their management in agricultural and horticultural crops; Mani M., Shivaraju C., Eds.; Springer India: New Delhi, India, 2016; pp 239–653.

[ref4] DeCastella F.; French C. Mealybug (*Dactylopius longispinus*), a potential vine pest. J. Dep. Agric., Victoria, Aust. 1929, 27, 427–433.

[ref5] Flanders S. E. Biological control of the longtailed mealybug *Pseudococcus longispinus*. J. Econ. Entomol. 1940, 33, 754–759. 10.1093/jee/33.5.754.

[ref6] Daane K. M.; Almeida R. P.; Bell V. A.; Walker J. T.; Botton M.; Fallahzadeh M.; Mani M.; Miano J. L.; Sforza R.; Walton V. M.; Zaviezo T. Biology and management of mealybugs in vineyards. In Arthropod management in vineyards: Pests, approaches, and future directions; Bostanian N., Vincent C., Isaacs R., Eds.; Springer: Dordrecht, Netherlands, 2012; pp 271–307.

[ref7] Targioni-Tozzetti A. Studii sulle Cocciniglie. Mem. Soc. ital. sci. nat. Mus. civ. stor. nat. Milano 1867, 3, 1–87.

[ref8] Pellizzari G.; Germain J. F. Scales (Hemiptera, Superfamily Coccoidea). Chapter 9.3. BioRisk 2010, 4, 475–510. 10.3897/biorisk.4.45.

[ref9] La Notte P.; Buzkan N.; Choueiri E.; Minafra A.; Martelli G. P. Acquisition and transmission of grapevine virus A by the mealybug *Pseudococcus longispinus*. J. Plant Pathol. 1997, 79, 79–85.

[ref10] Petersen C. L.; Charles J. G. Transmission of grapevine leafroll-associated closteroviruses by *Pseudococcus longispinus* and *P. calceolariae*. Plant Pathol. 1997, 46, 509–515. 10.1046/j.1365-3059.1997.d01-44.x.

[ref11] Golino D. A.; Sim S. T.; Gill R.; Rowhani A. California mealybugs can spread grapevine leafroll disease. Calif. Agric. 2002, 56, 196–201. 10.3733/ca.v056n06p196.

[ref12] IVIA-Instituto Valenciano de Investigaciones Científicas. Gestión integrada de Plagas y Enfermedades en Caqui. : http://gipcaqui.ivia.es/ (accessed Oct 10, 2023).

[ref13] Navarro-Llopis V.; Primo J.; Navarro I.; Vacas S. Seguimiento y distribución del cotonet de Sudáfrica *Delotococcus aberiae* De Lotto (Hemiptera: Pseudococcidae) en la Comunidad Valenciana mediante trampas cebadas con su feromona sexual. Phytoma España 2019, 311, 56–61.

[ref14] Franco J. C.; Cocco A.; Lucchi A.; Mendel Z.; Suma P.; Vacas S.; Mansour R.; Navarro-Llopis V. Scientific and technological developments in mating disruption of scale insects. Entomol. Gen. 2022, 42, 251–273. 10.1127/entomologia/2021/1220.

[ref15] Millar J. G.; Moreira J. A.; McElfresh S.; Daane K. M.; Freund A. S. Sex pheromone of the longtailed mealybug: a new class of monoterpene structure. Org. Lett. 2009, 11, 2683–2685. 10.1021/ol802164v. 19449888

[ref16] Williams D. J.; Granara D. W. Mealybugs of Central and South America; CAB International: Wallingford, UK, 1992; p 635.

[ref17] Granara D. W.; Gonzáles P. Taxonomic review of *Pseudococcus* Westwood (Hemiptera: Pseudococcidae) from Central and South America with descriptions of new species. Insecta Mundi 2018, 673, 1–117.

[ref18] Moghaddam M. A review of the mealybugs (Hemiptera: Coccoidea: Pseudococcidae, Putoidae and Rhizoecidae) of Iran, with descriptions of four new species and three new records for the Iranian fauna. Zootaxa 2013, 3632 (1), 1–107. 10.11646/zootaxa.3632.1.1. 25325093

[ref19] Zou Y.; Millar J. G. Improved synthesis of the pheromone of the longtailed mealybug. Synlett 2010, 2010, 2319–2321. 10.1055/s-0030-1258025.

[ref20] Vacas S.; Navarro I.; Marzo J.; Navarro-Llopis V.; Primo J. Sex pheromone of the invasive mealybug citrus pest, *Delottococcus aberiae* (Hemiptera: Pseudococcidae). A new monoterpenoid with a necrodane skeleton. J. Agric. Food Chem. 2019, 67, 9441–9449. 10.1021/acs.jafc.9b01443. 31381358

[ref21] Conia J. M. No. 515-Sur la preparation de cyclopentenones par adtion de l’acide polphosphorique sur les esters d’acides α-ethyleniques. 1 Part: Aspects pratiques. Bull. Soc. Chim. Fr. 1970, 8–9, 2981–2991.

[ref22] Tabata J.; Ichiki R. T.; Moromizato C.; Mori K. Sex pheromone of a coccoid insect with sexual and asexual lineages: fate of an ancestrally essential sexual signal in parthenogenetic females. J. R. Soc., Interface 2017, 14, 20170027 10.1098/rsif.2017.0027. 28250102 PMC5378144

[ref23] Majetich G.; L Grove J. Synthesis of 6-Hydroxyisochromenes and 6-Hydroxyisocoumarins from 3-Ethoxycyclohex-2-en-1-one. Heterocycles 2012, 84, 963–982. 10.3987/com-11-s(p)78.

[ref24] Aggarwal V. K.; Belfield A. J. Catalytic asymmetric Nazarov reactions promoted by chiral Lewis acid complexes. Org. Lett. 2003, 5, 5075–5078. 10.1021/ol036133h. 14682768

[ref25] Curran T. T.; Hay D. A.; Koegel C. P.; Evans J. C. The preparation of optically active 2-cyclopenten-1,4-diol derivatives from furfuryl alcohol. Tetrahedron 1997, 53, 1983–2004. 10.1016/S0040-4020(96)01169-6.

[ref26] Sullivan N. J.; Bell V. A.; Butler R. C.; Wallis R.; Ramesh R.; Reddy D. S.; Twidle A. M.; Bunn B.; Unelius C. R.; Manning L. A. M.; Suckling D. M. Developing a mealybug pheromone monitoring tool to enhance IPM practices in New Zealand vineyards. J. Pestic. Sci. 2023, 96, 29–39. 10.1007/s10340-022-01504-5.

[ref27] Thomas Y.; Bethenod M. T.; Pelozuelo L.; Frérot B.; Bourguet D. Genetic isolation between two sympatric host-plant races of the European corn borer, *Ostrinia nubilalis* Hübner. I. Sex pheromone, moth emergence timing, and parasitism. Evolution 2003, 57, 261–273. 10.1111/j.0014-3820.2003.tb00261.x. 12683523

[ref28] Zada A.; Dunkelblum E.; Assael F.; Harel M.; Cojocaru M.; Mendel Z. Sex pheromone of the vine mealybug, *Planococcus ficus* in Israel: occurrence of a second component in a mass-reared population. J. Chem. Ecol. 2003, 29, 977–988. 10.1023/A:1022944119077. 12775156

[ref29] Ferris G. F. The California species of mealy bugs; Stanford University Publications. University Series: Palo Alto, CA, 1918; pp 1–78.

[ref30] Frérot B.; Leppik E.; Groot A. T.; Unbehend M.; Holopainen J. K. Chemical signatures in plant-insect interactions. In Advances in Botanical Research; Sauvion N., Thiéry D., Calatayud P. A., Eds.; Academic Press: USA, 2017; Vol. 81, pp 139–177. 10.1016/bs.abr.2016.10.003.

[ref31] Daane K. M.; Cooper M. L.; Mercer N. H.; Hogg B. N.; Yokota G. Y.; Haviland D. R.; Welter S. C.; Cave F. E.; Sial A. A.; Boyd E. A.; Burks C. Pheromone Deployment Strategies for Mating Disruption of a Vineyard Mealybug. J. Econ. Entomol. 2021, 114, 2439–2451. 10.1093/jee/toab198. 34694405 PMC8648387

[ref32] Ballesteros C.; Romero A.; Castro M. C.; Miranda S.; Bergmann J.; Zaviezo T. Mating Disruption of *Pseudococcus calceolariae* (Maskell) (Hemiptera, Pseudococcidae) in Fruit Crops. Insects 2021, 12, 343 10.3390/insects12040343. 33924297 PMC8069303

[ref33] Teshiba M.; Shimizu N.; Sawamura N.; Narai Y.; Sugie H.; Sasaki R.; Tabata J.; Tsutsumi T. Use of a sex pheromone to disrupt the mating of *Planococcus kraunhiae* (Kuwana) (Hemiptera: Pseudococcidae). Jpn. J. Appl. Entomol. Zool. 2009, 53, 173–180. 10.1303/jjaez.2009.173.

[ref34] Cocco A.; Lentini A.; Serra G. (2014). Mating disruption of *Planococcus ficus* (Hemiptera: Pseudococcidae) in vineyards using reservoir pheromone dispensers. J. Insect Sci. 2014, 14 (1), 1–8. 10.1093/jisesa/ieu006. 25347835 PMC5443473

[ref35] Walton V. M.; Daane K. M.; Bentley W. J.; Millar J. G.; Larsen T. E.; Malakar-Kuenen R. Pheromone-based mating disruption of *Planococcus ficus* (Hemiptera: Pseudococcidae) in California vineyards. J. Econ. Entomol. 2006, 99, 1280–1290. 10.1093/jee/99.4.1280. 16937683

[ref36] Tabata J.; Teshiba M.; Shimizu N.; Sugie H. Mealybug mating disruption by a sex pheromone derived from lavender essential oil. J. Essent. Oil Res. 2015, 27, 232–237. 10.1080/10412905.2015.1007219.

[ref37] Tacoli F.; Bell V. A.; Cargnus E.; Pavan F. Insecticidal activity of natural products against vineyard mealybugs (Hemiptera: Pseudococcidae). Crop Prot. 2018, 111, 50–57. 10.1016/j.cropro.2018.04.020.

[ref38] Waterworth R. A.; Redak R. A.; Millar J. G. Pheromone-baited traps for assessment of seasonal activity and population densities of mealybug species (Hemiptera: Pseudococcidae) in nurseries producing ornamental plants. J. Econ. Entomol. 2011, 104, 555–565. 10.1603/EC10317. 21510204

[ref39] Waterworth R. A.; Wright I. M.; Millar J. G. Reproductive biology of three cosmopolitan mealybug (Hemiptera: Pseudococcidae) species, *Pseudococcus longispinus, Pseudococcus viburni*, and *Planococcus ficus*. Ann. Entomol. Soc. Am. 2011, 104, 249–260. 10.1603/AN10139.

