# Supplementary material for: New Monoterpenoid as the Sex Pheromone of Spanish Populations of the Longtailed Mealybug Pseudococcus Longispinus (Hemiptera: Pseudococcidae)
Source: J Agric Food Chem. 2024 May 21;72(22):12478–88. doi: 10.1021/acs.jafc.4c00921 (PMC11157542; doi:10.1021/acs.jafc.4c00921)
Supplement: Supplementary file 1 — jf4c00921_si_001.pdf [file jf4c00921_si_001.pdf]

Manuscript prepared for Journal of Agricultural and Food Chemistry

Title: A new monoterpenoid as sex pheromone of Spanish populations of the longtailed mealybug Pseudococcus longispinus (Hemiptera:Pseudococcidae)

Sandra Vacas,[1]* Ismael Navarro Fuertes [2]*, Víctor García-García,[2] Javier Marzo,[3] Antonio Abad Somovilla,[2] Jaime Primo[1], Vicente Navarro-Llopis [1]

[1] CEQA-Instituto Agroforestal del Mediterráneo, Universitat Politècnica de València, Camino de Vera s/n, edificio 6C-5ª planta, 46022 Valencia (Valencia), Spain. [2] Universitat de València, Facultat de Químiques, Departamento de Química Orgánica, Dr. Moliner 50, 46100 Burjassot (Valencia), Spain. [3] Ecología y Protección Agrícola SL, Pol. Ind. Ciutat de Carlet, 46240 Carlet (Valencia), Spain.

*Correspondence to: Sandra Vacas. CEQA-Instituto Agroforestal del Mediterráneo, Universitat Politècnica de València. Camino de Vera s/n, edificio 6C-5ª planta, 46022-Valencia, Spain. Email: sanvagon@ceqa.upv.es; phone: +34963879058. Ismael Navarro. Universitat de València, Facultat de Químiques, Departamento de Química Orgánica, Dr. Moliner 50, 46100, Burjassot (Valencia), Spain. Email: isnafuer@uv.es; phone: +34963543886.

Supporting Information

Figures S1-S24: ^{1}H NMR and ^{13}C NMR spectra of the synthetic compounds.

Figure S25: GC-MS spectrum of 2-(1,5-dimethyl-4-cyclopent-2-en-1-yl)ethanol.

Figure S26: Multiplicity-edited HSQC spectra of isolated sex pheromone of Spanish populations of *P. longispinus*

Figure S27. Multiplicity-edited HSQC spectra of compound **3**.

Figure S28. ORTEP diagram for compound **15**. Thermal ellipsoids are shown with 50% probability. For details, see CCDC 2327985 (Cambridge Crystallographic Data Centre).

Figure S29. Mean (±SE) number of males captured per trap and week in traps baited with virgin females and those baited with 2-(1,5,5-trimethylcyclopent-2-en-1-yl)ethyl acetate **1**. Differences were significant by ANOVA (Tukey HSD test at $P < 0.05$).

Table. Crystal data and structure refinement for compound **15**.

Figure S1. ^{1}H NMR spectrum of **5**.

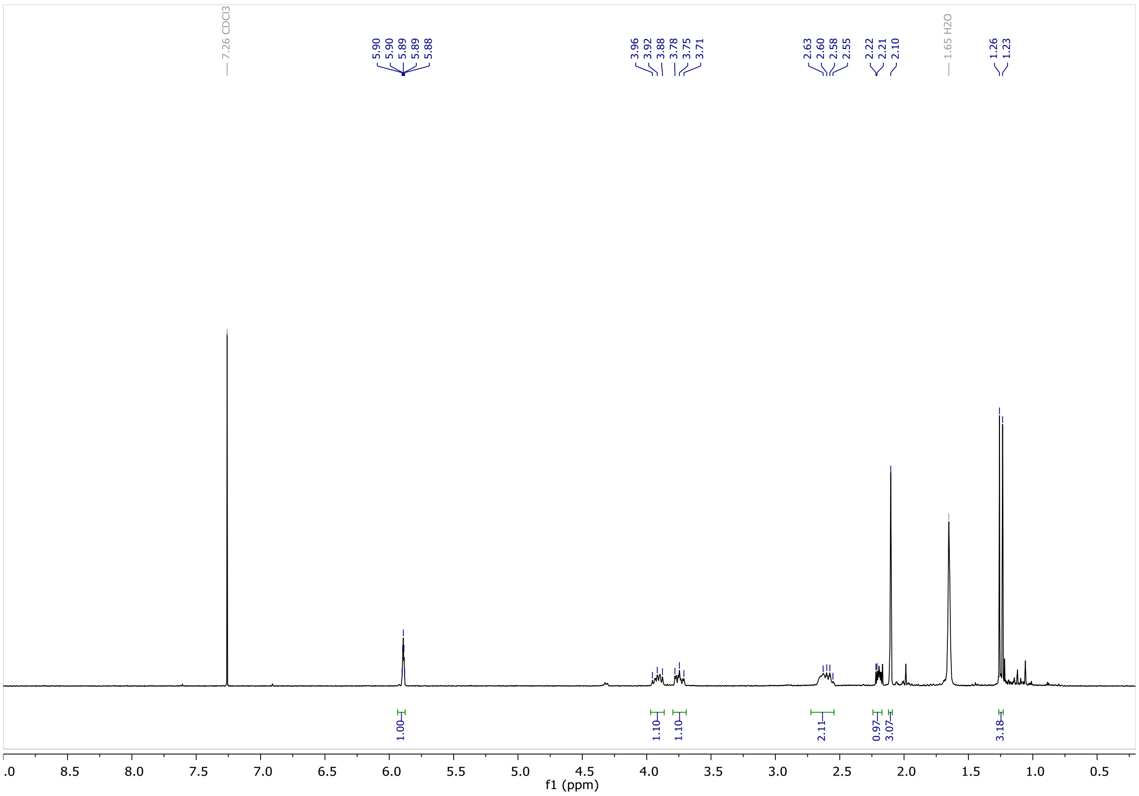

Figure S2. ^{13}C NMR spectrum of **5**.

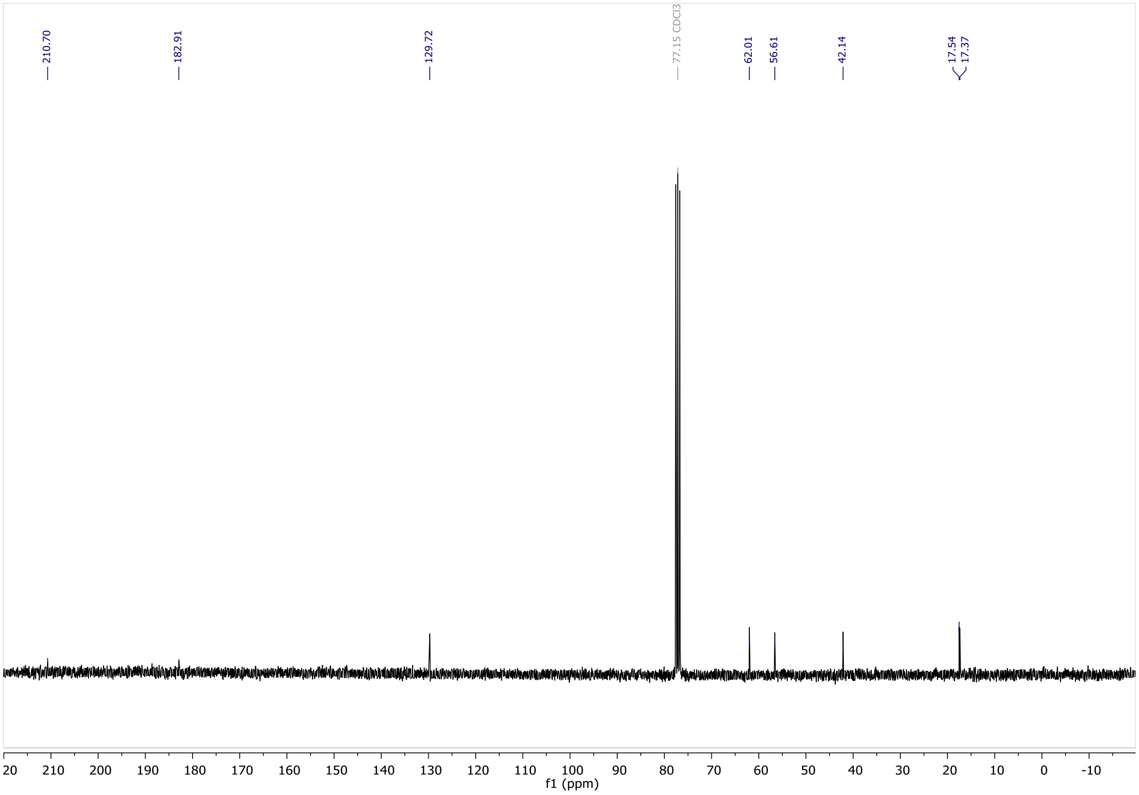

Figure S3. ^{1}H NMR spectrum of **6.**

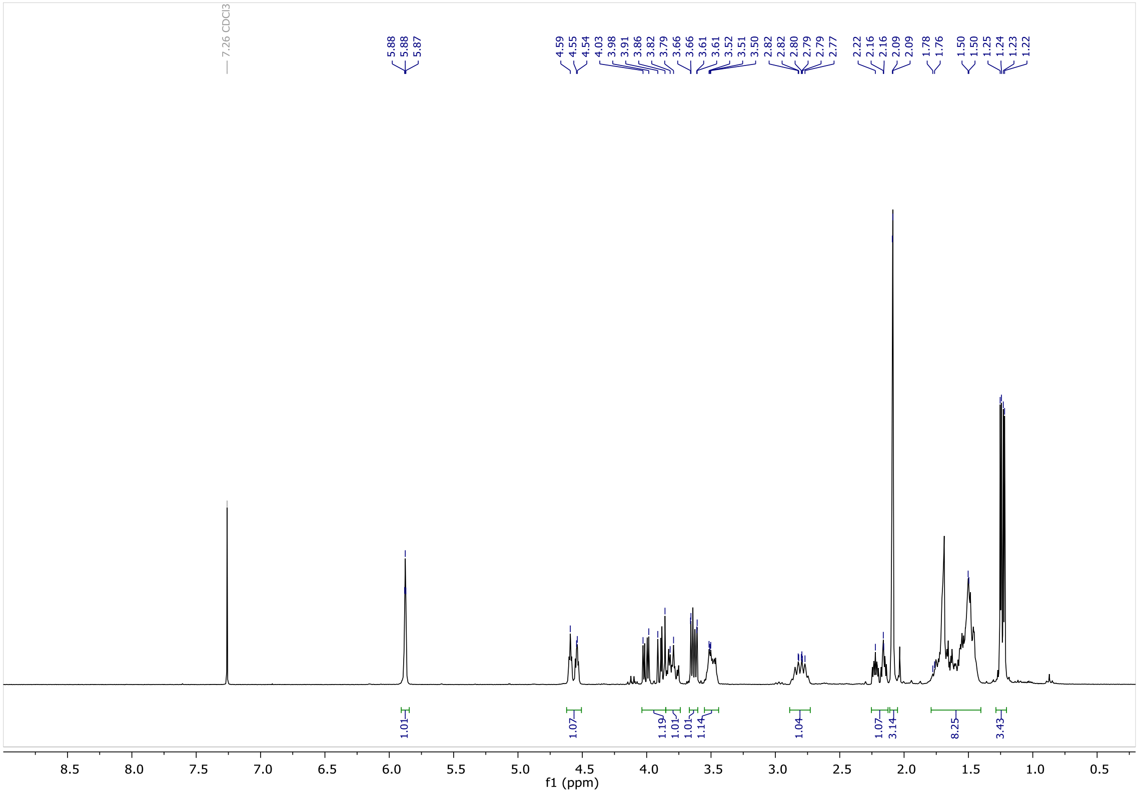

Figure S4. ^{13}C NMR spectrum **6**.

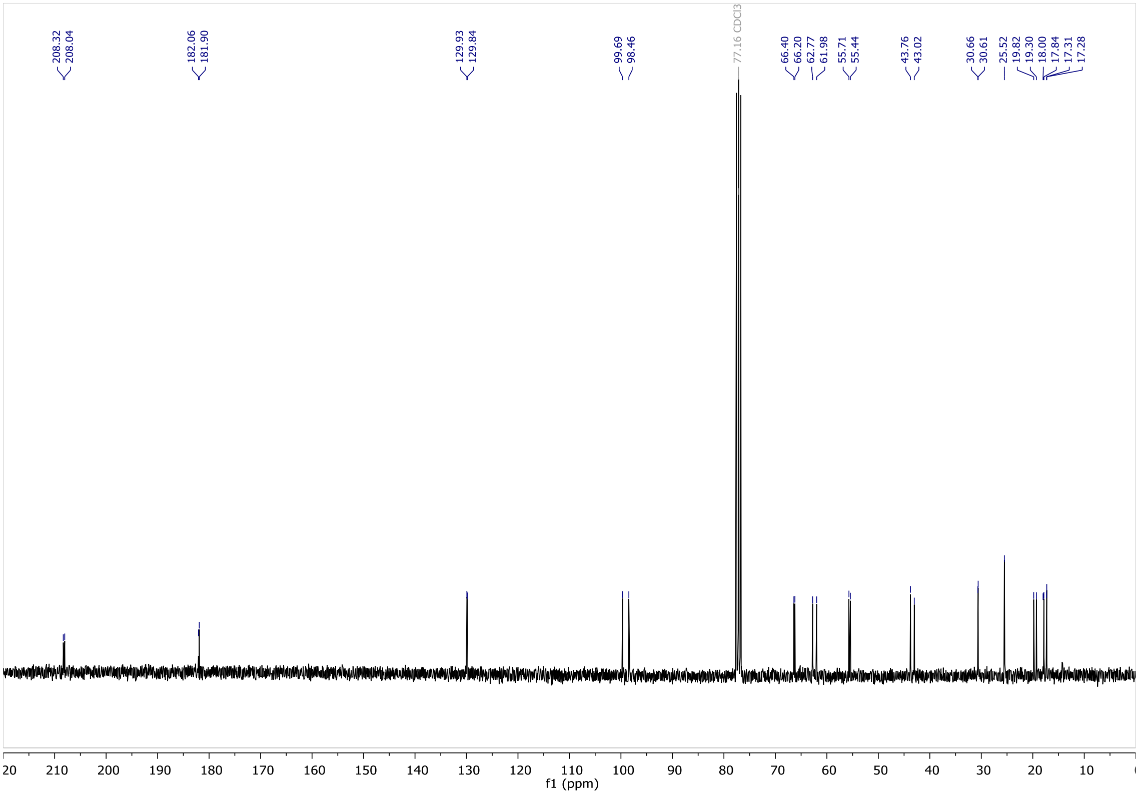

Figure S5. ^{1}H NMR spectrum of **7.**

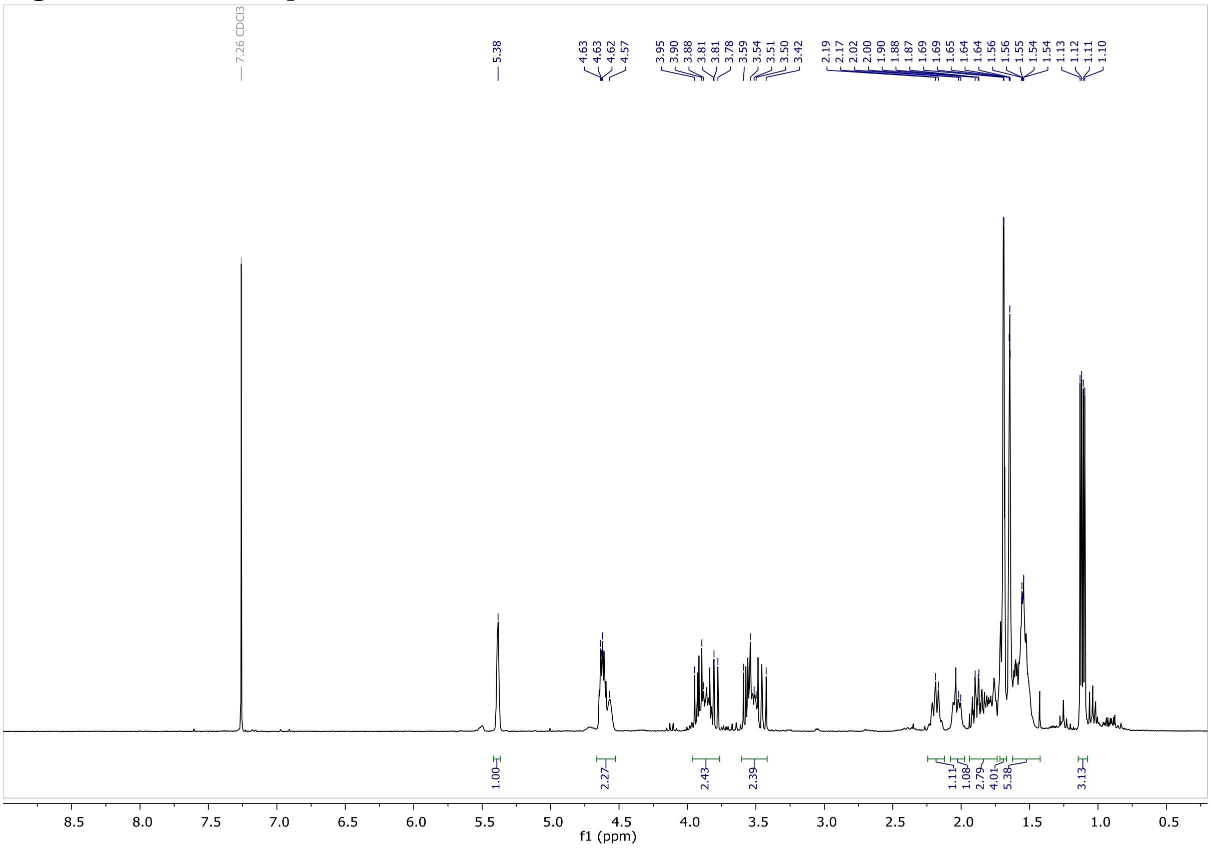

Figure S6. ^{13}C NMR spectrum **7**.

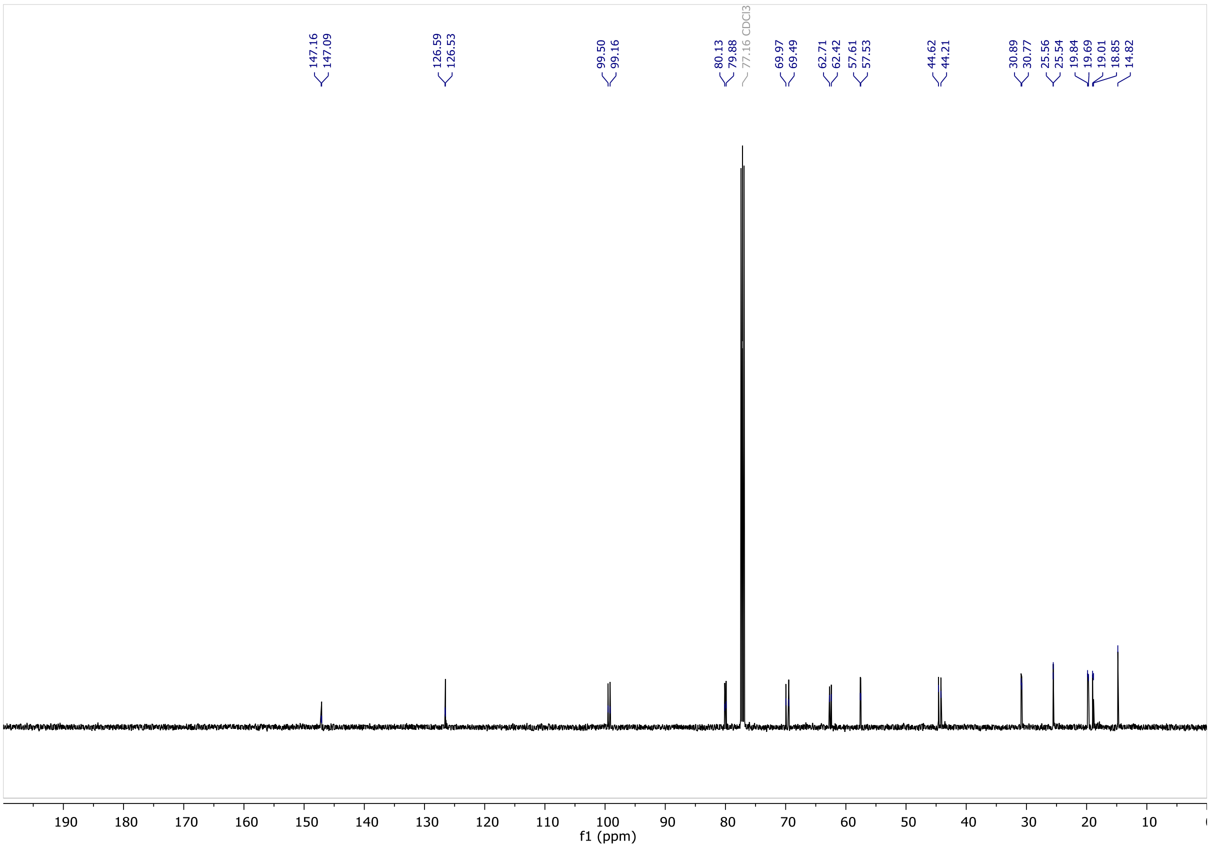

Figure S7. ^{1}H NMR spectrum of **8.**

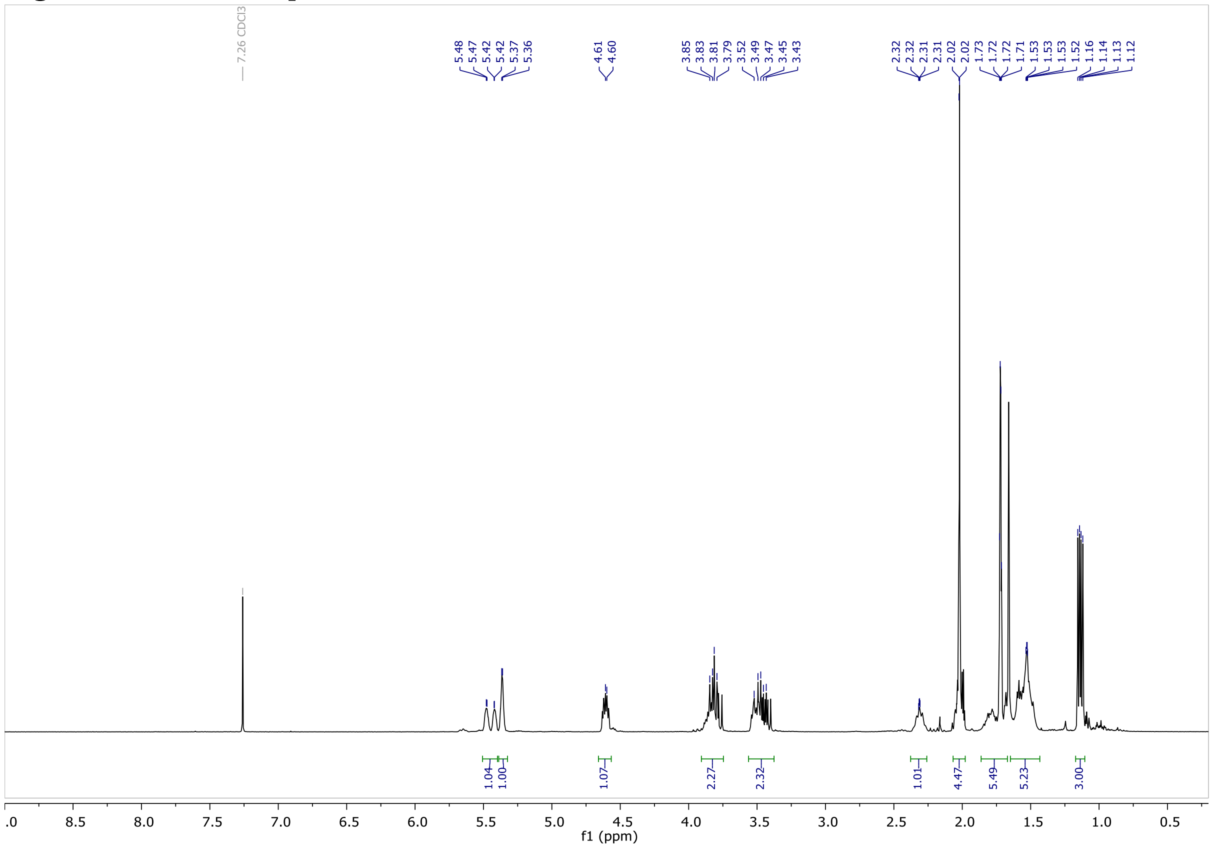

Figure S8. ^{13}C NMR spectrum **8**.

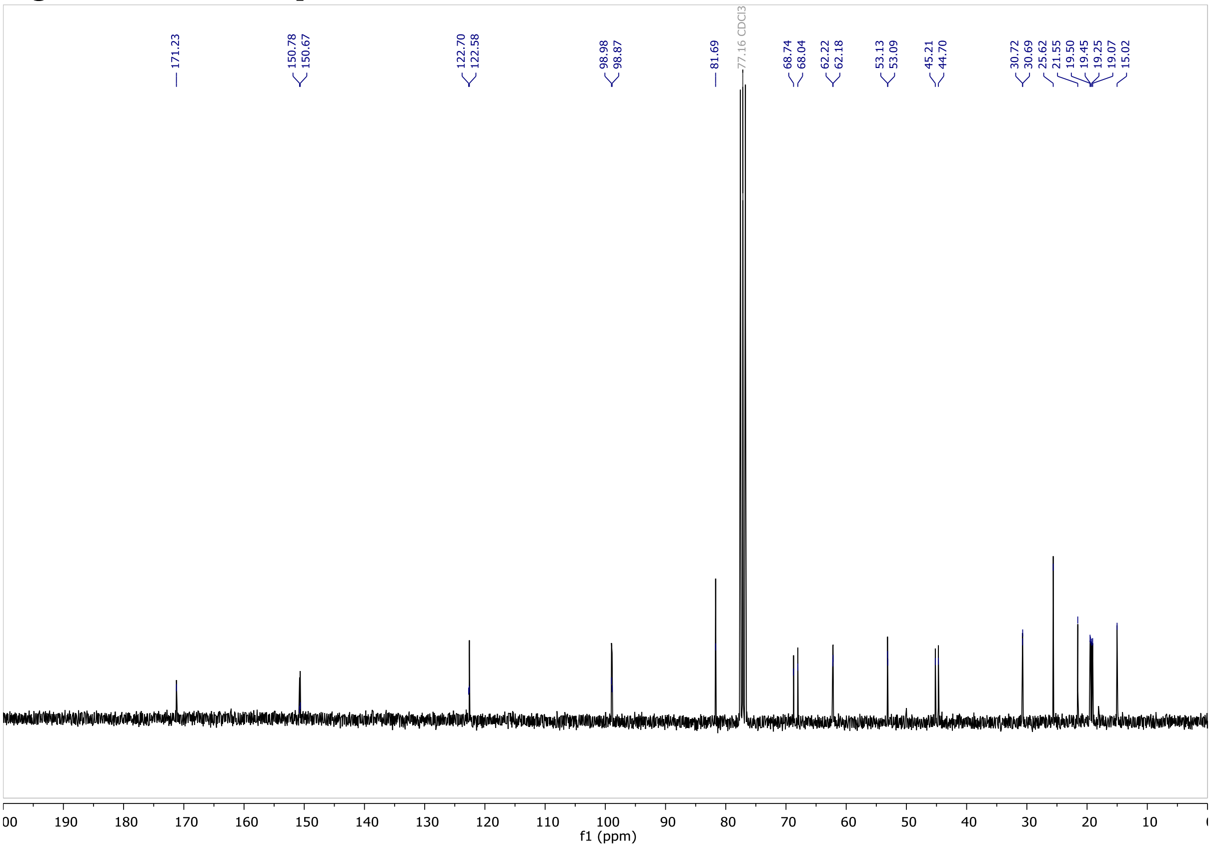

Figure S9. ^{1}H NMR spectrum of **9.**

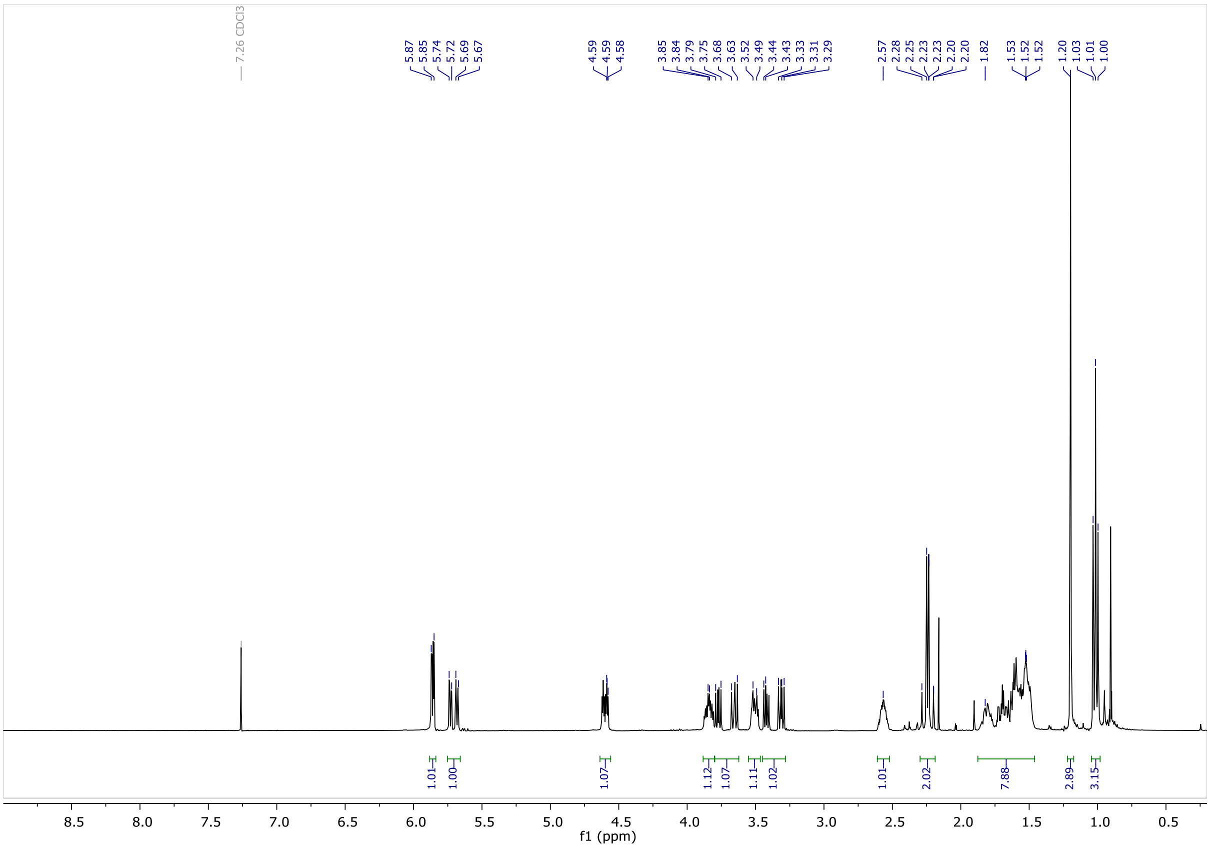

Figure S10. ^{13}C NMR spectrum **9**.

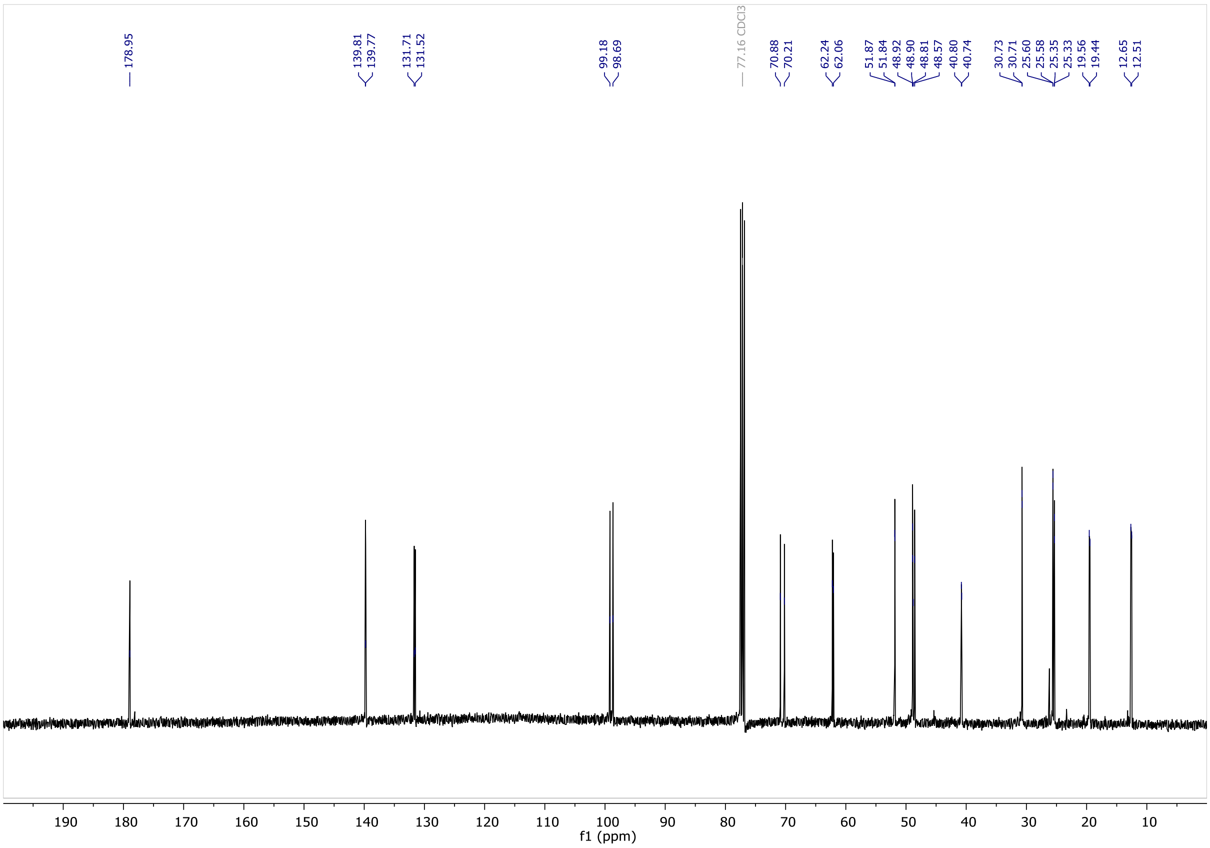

Figure S11. ^{1}H NMR spectrum of **10.**

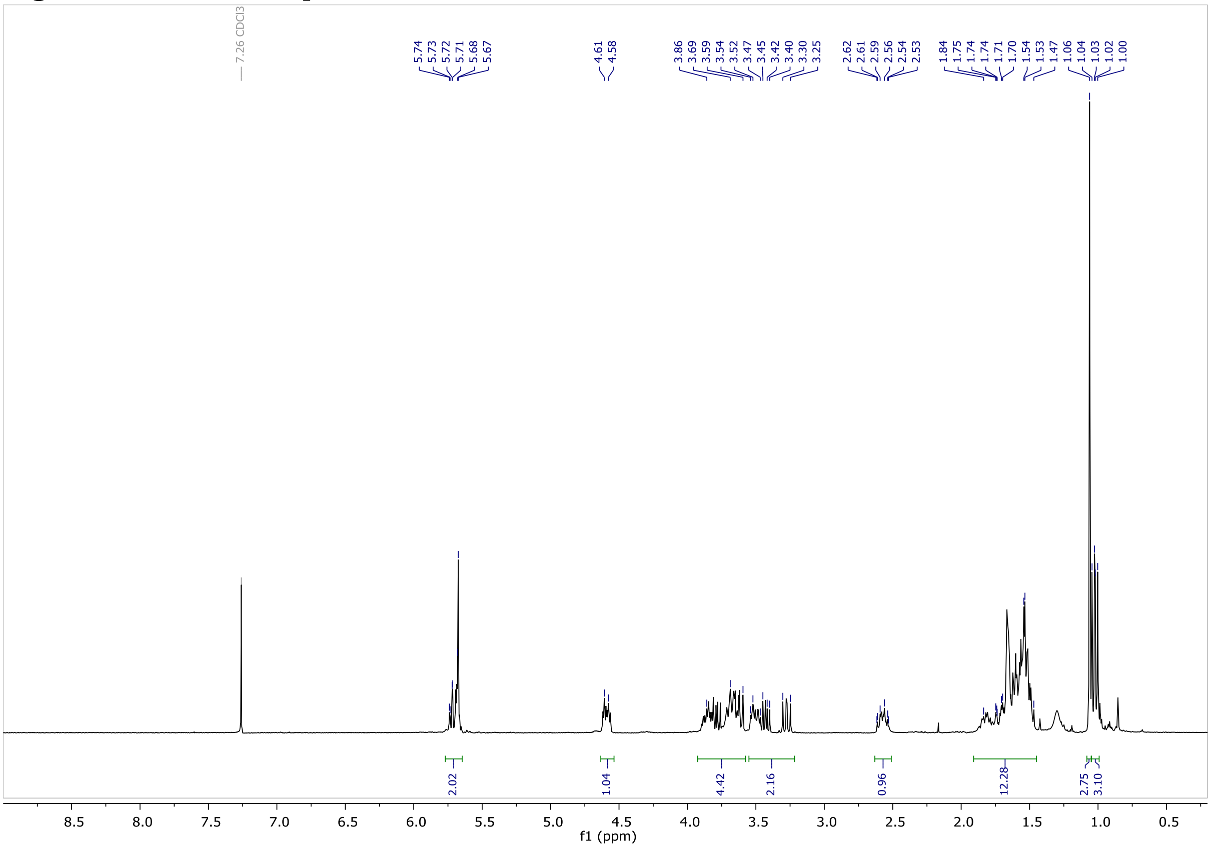

Figure S12. ^{13}C NMR spectrum **10**.

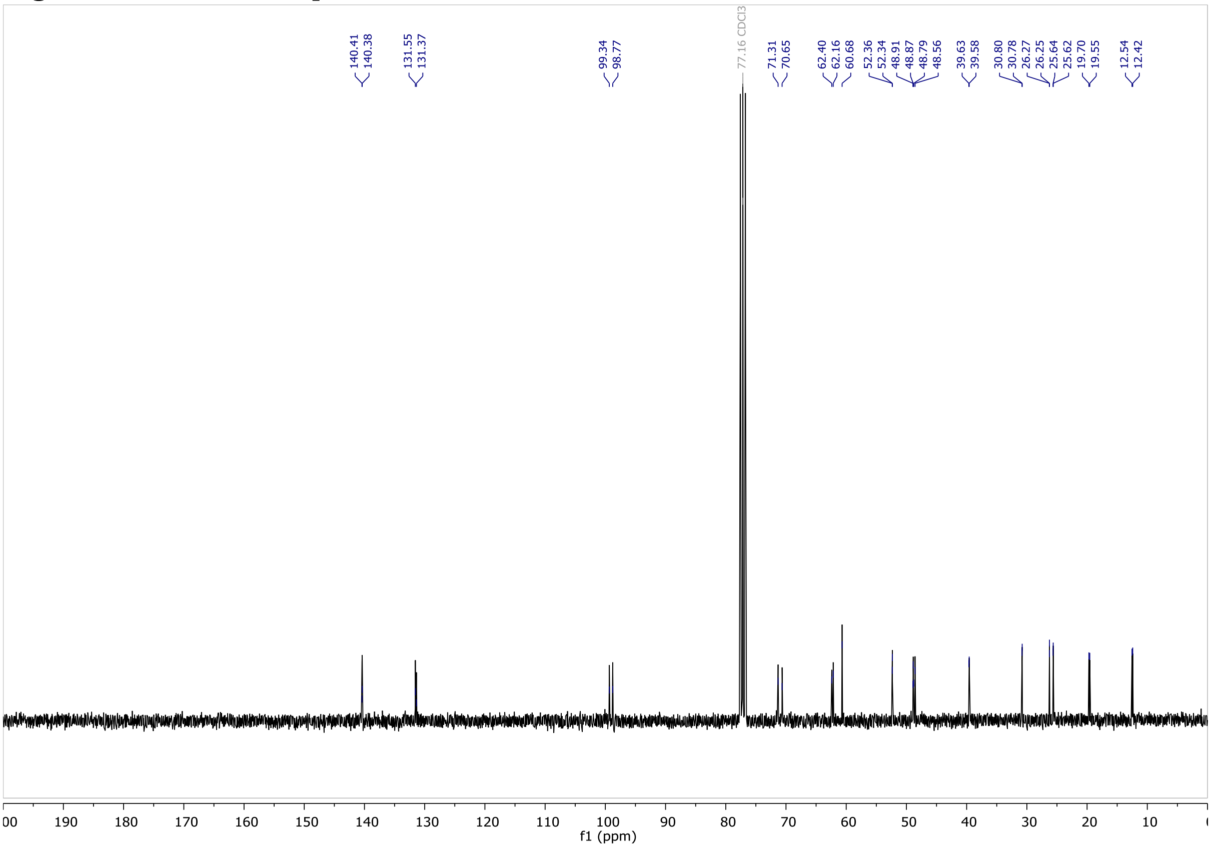

Figure S13. ^{1}H NMR spectrum of **14.**

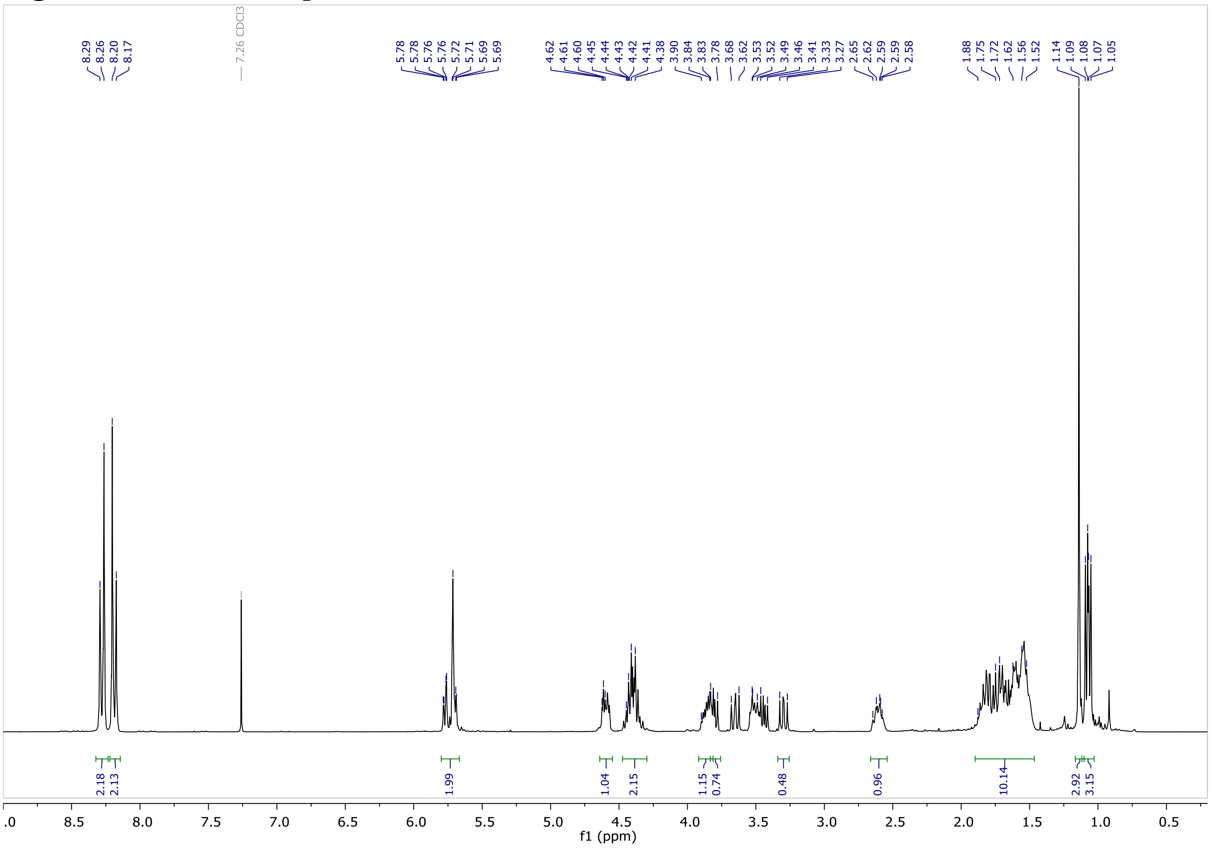

Figure S14. ^{13}C NMR spectrum **14**.

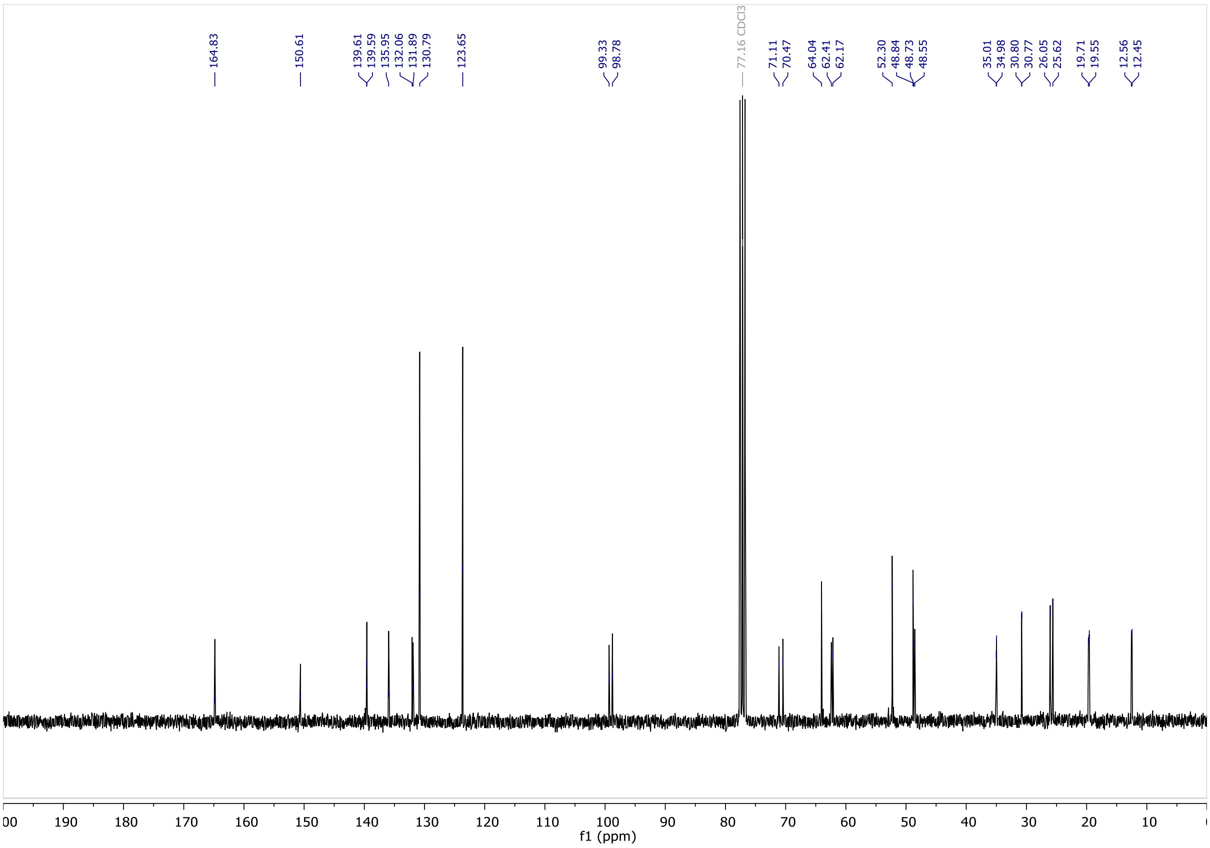

Figure S15. ^{1}H NMR spectrum of **15.**

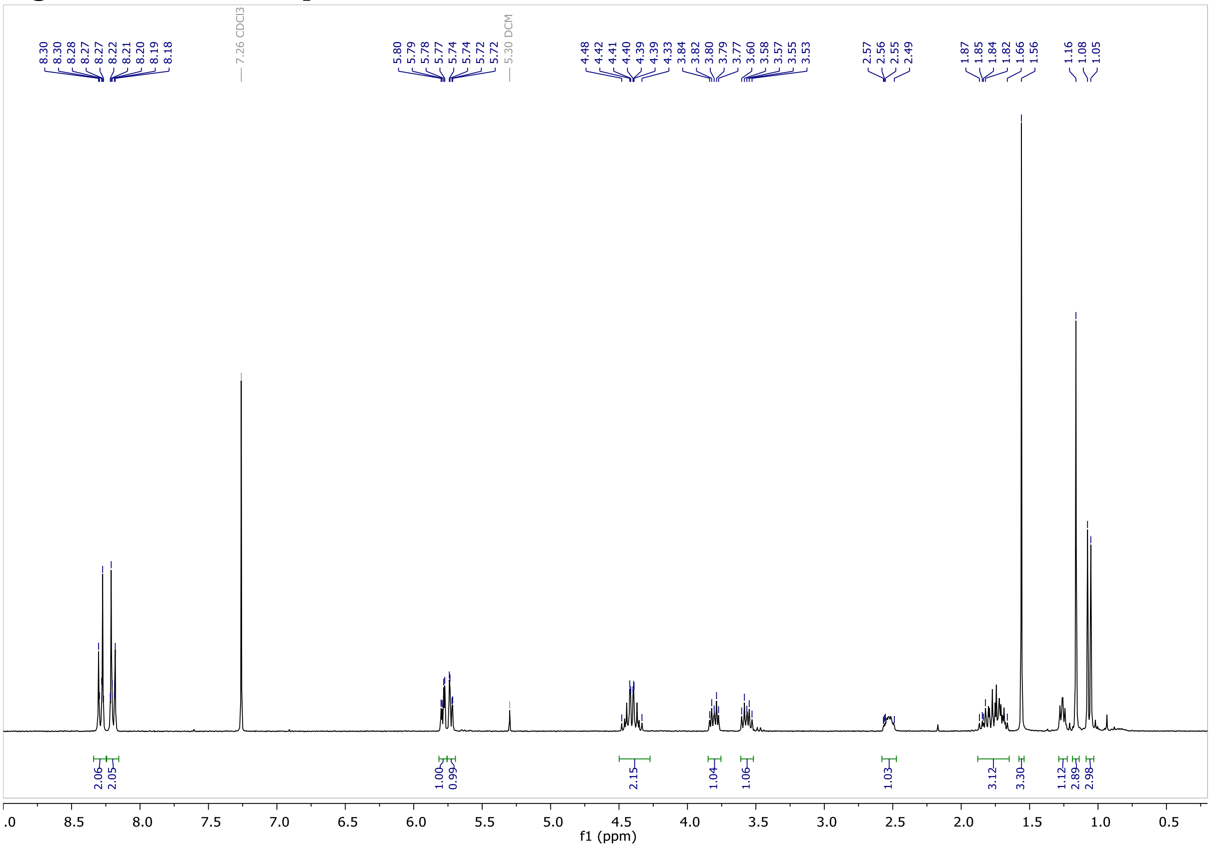

Figure S16. ^{13}C NMR spectrum **15**.

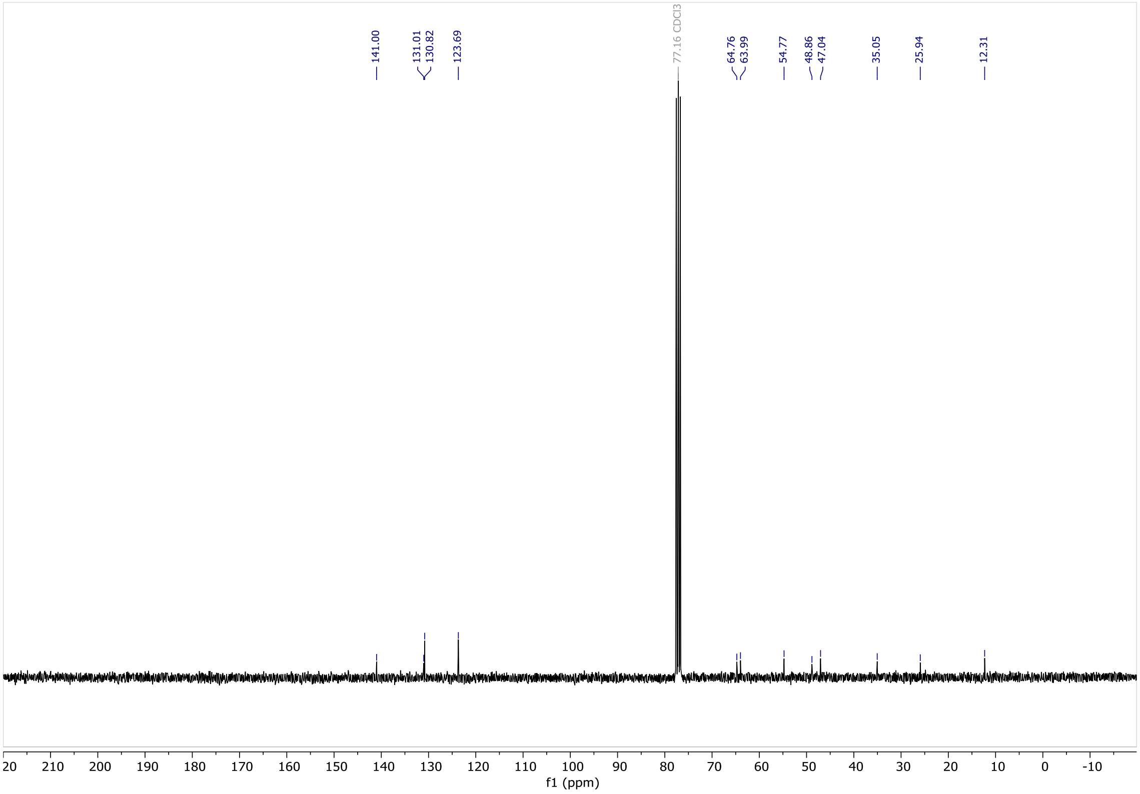

Figure S17. ^{1}H NMR spectrum of **11.**

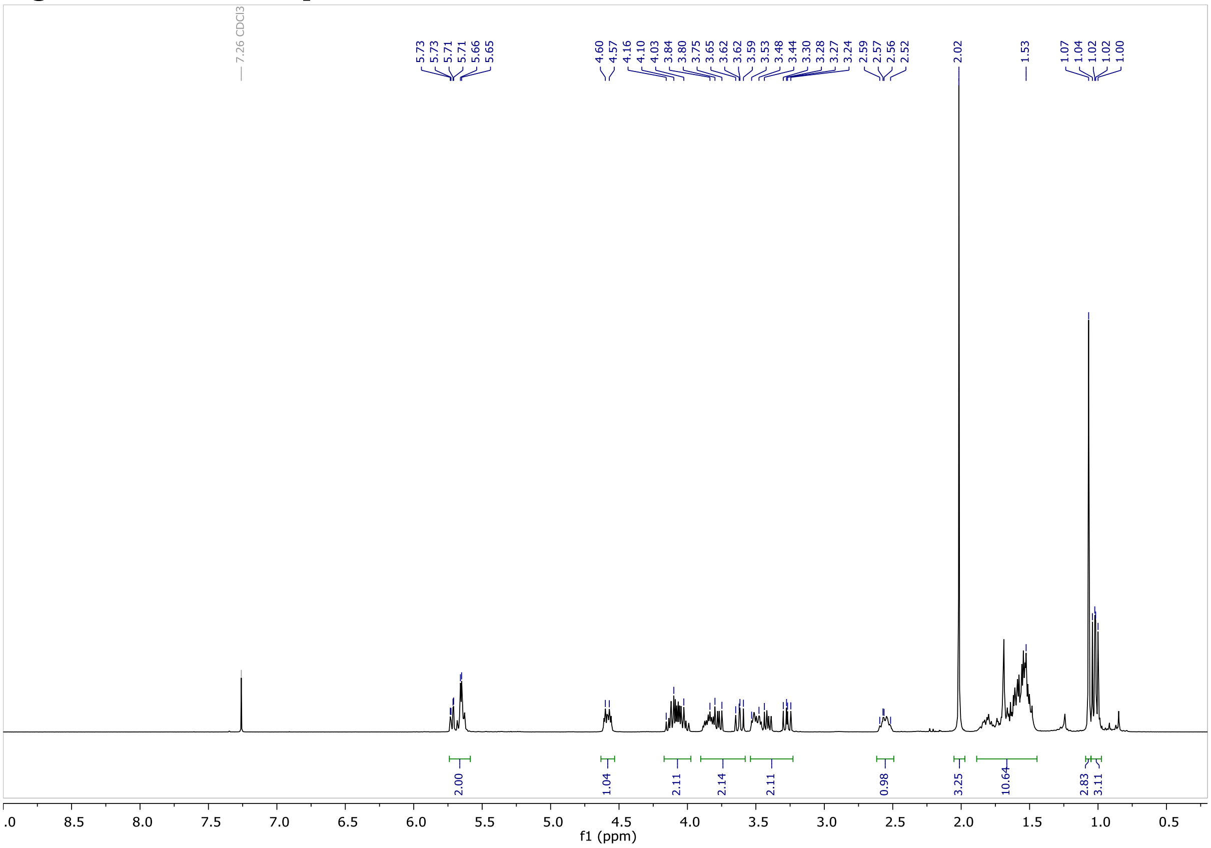

Figure S18. ^{13}C NMR spectrum **11**.

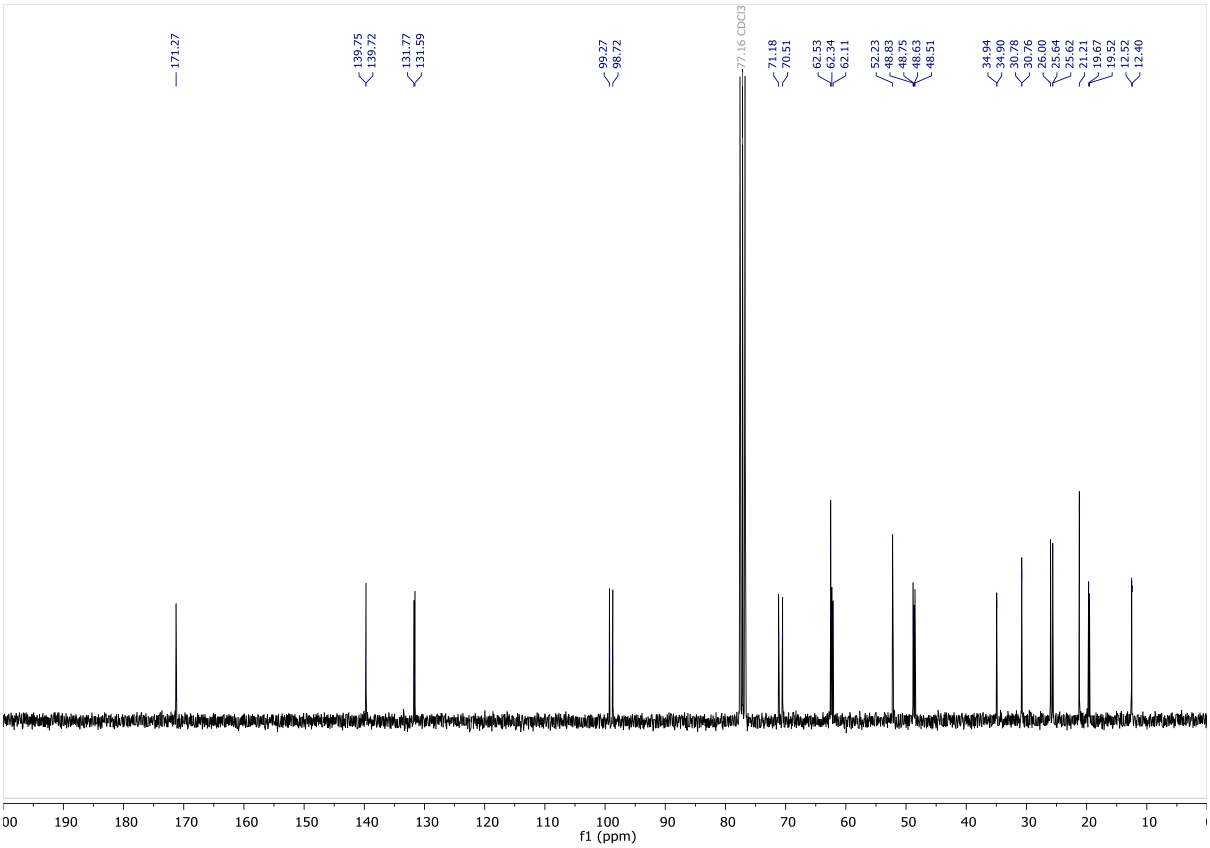

Figure S19. ^{1}H NMR spectrum of **12.**

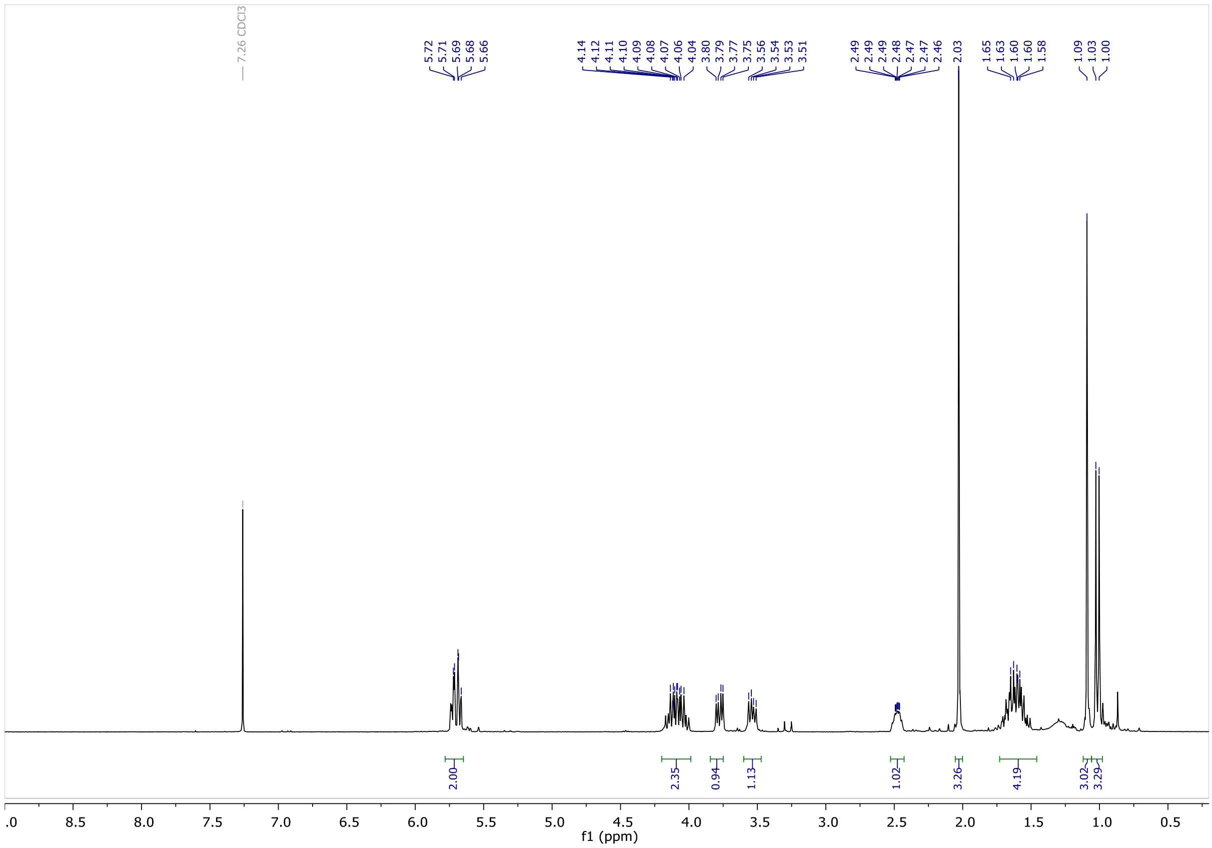

Figure S20. ^{13}C NMR spectrum **12**.

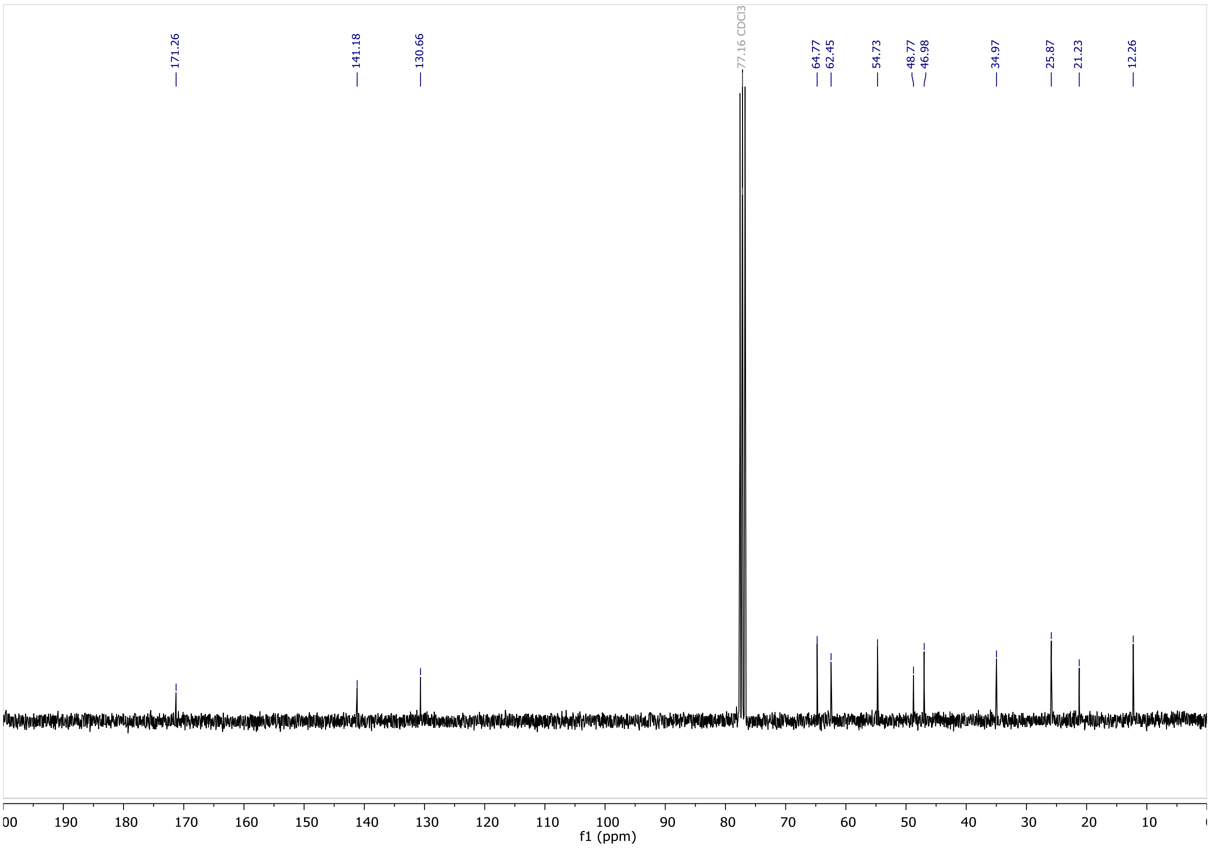

Figure S21. ^{1}H NMR spectrum of **13.**

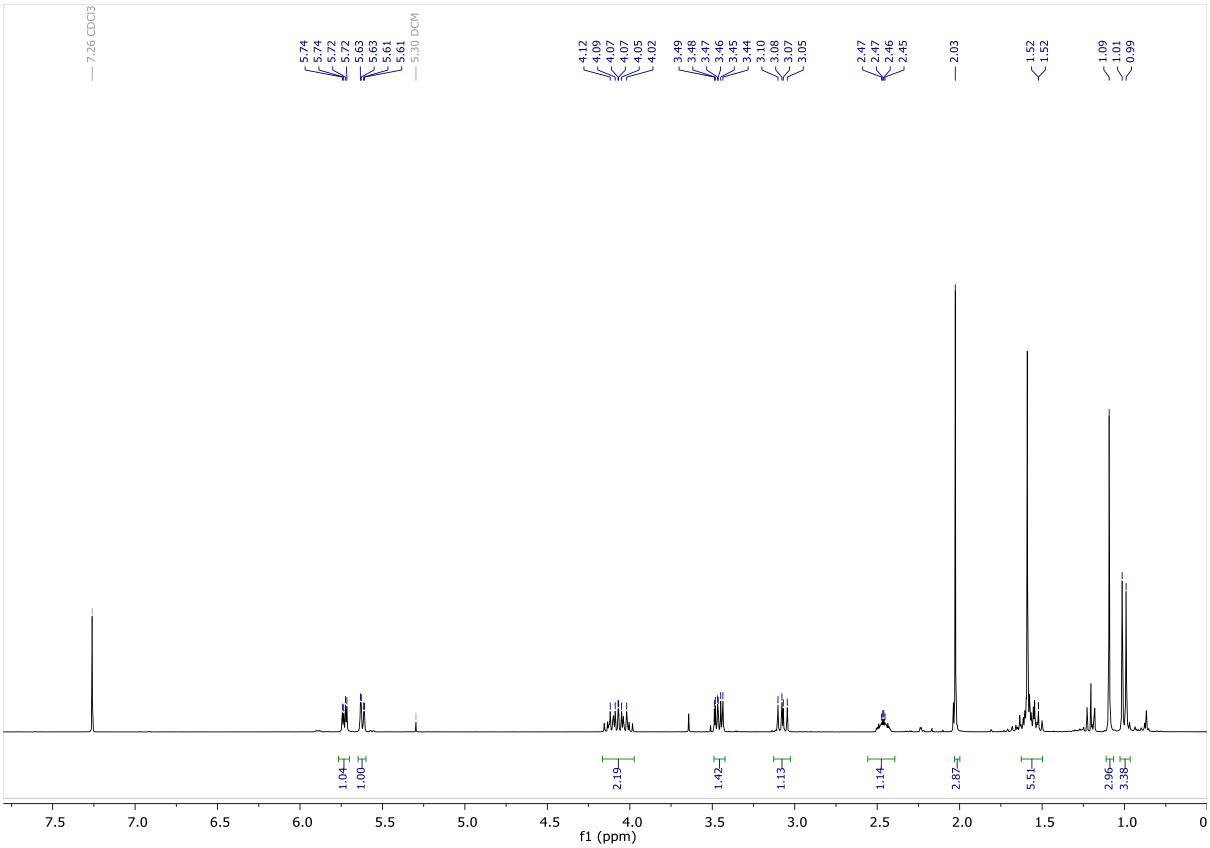

Figure S22. ^{13}C NMR spectrum **13**.

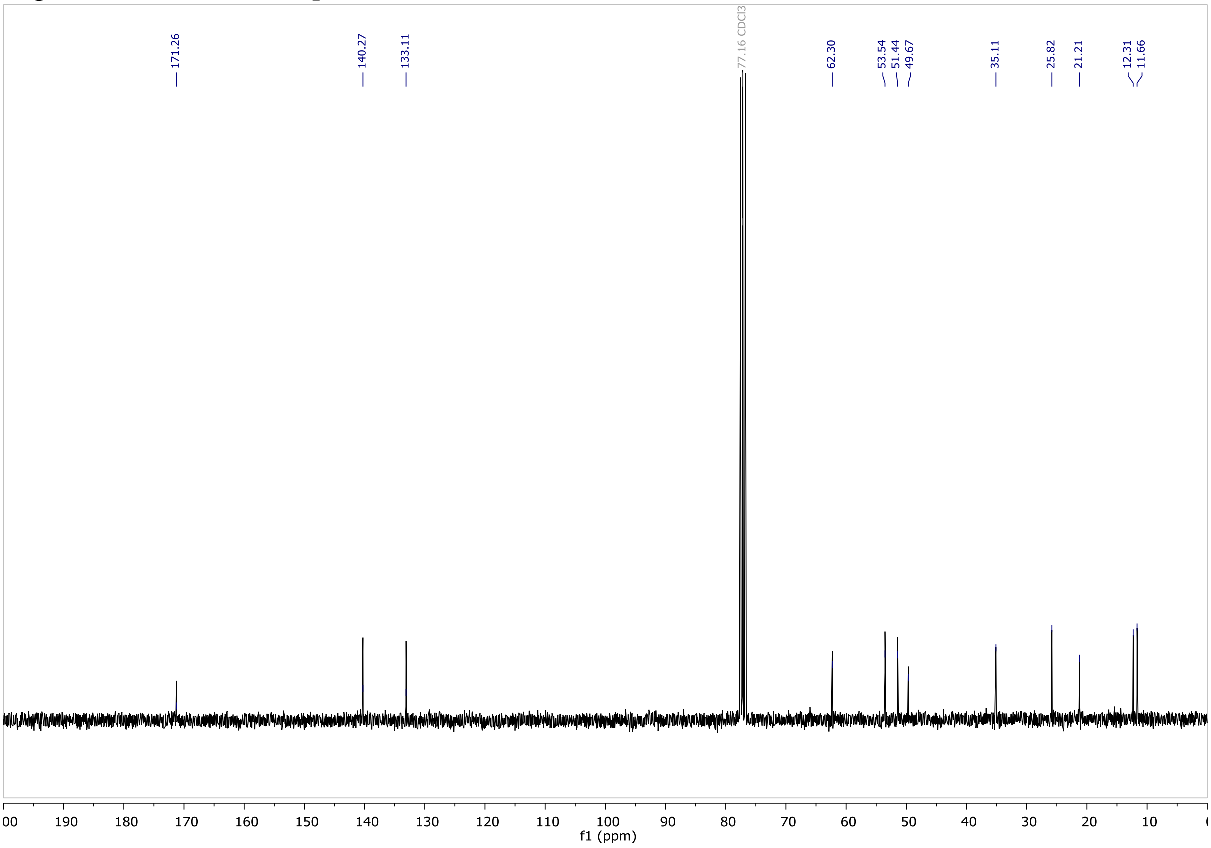

Figure S23. ^{1}H NMR spectrum of **3.**

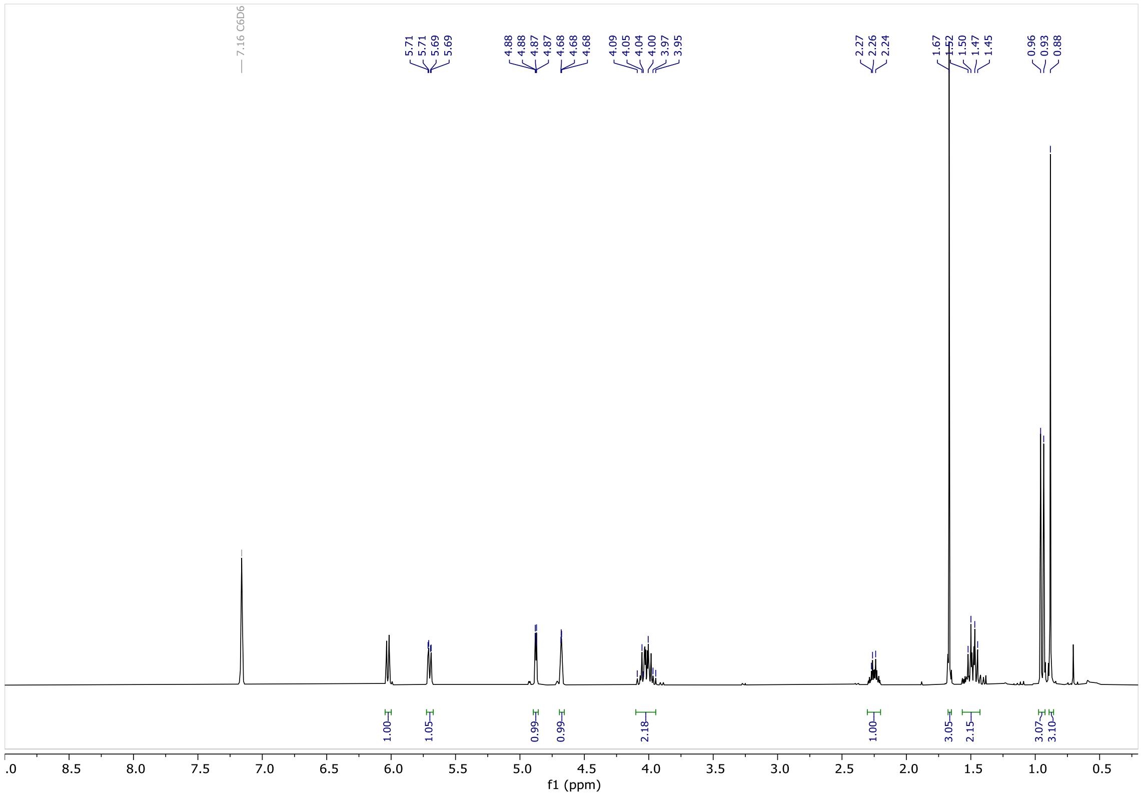

Figure S24. ^{13}C NMR spectrum **3**.

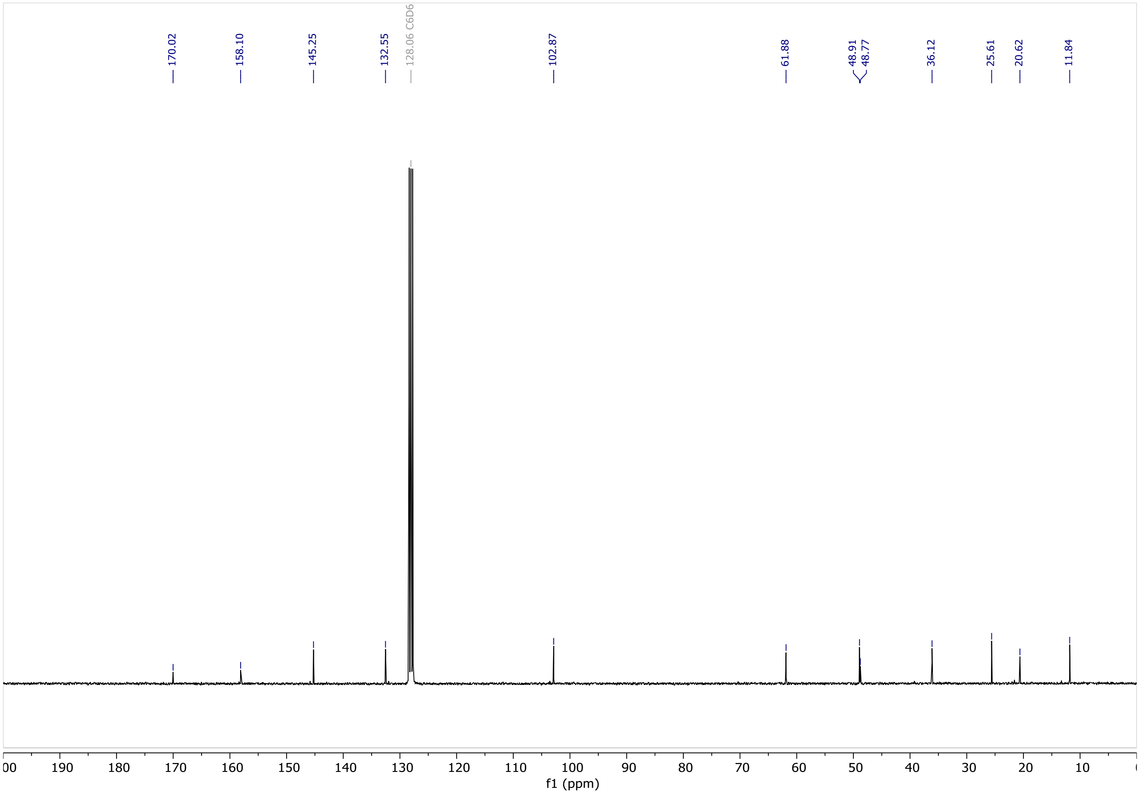

Figure S25. GC-MS spectrum of 2-(1,5-dimethyl-4-cyclopent-2-en-1-yl)ethanol

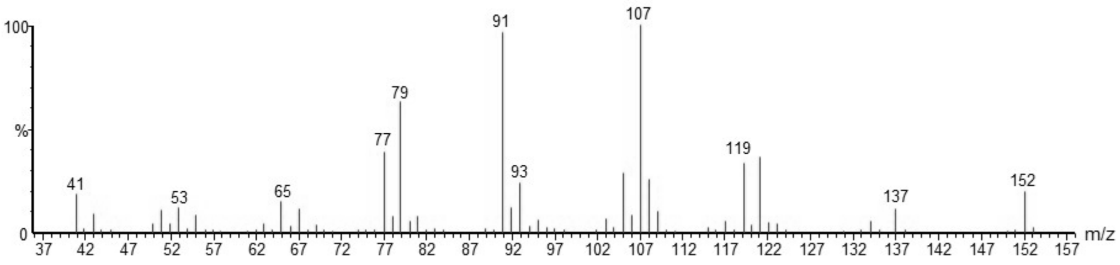

Figure S26. Multiplicity-edited HSQC spectra of isolated sex pheromone of Spanish populations of *P. longispinus*

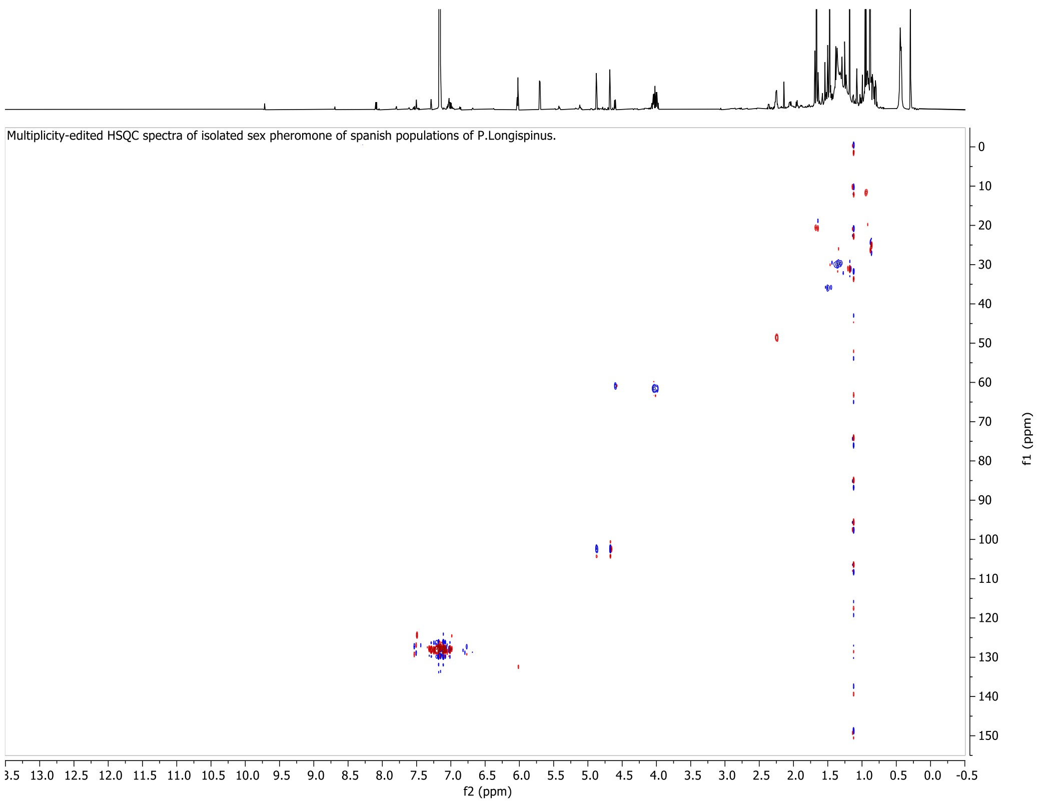

Figure S27. Multiplicity-edited HSQC spectra of compound **3**.

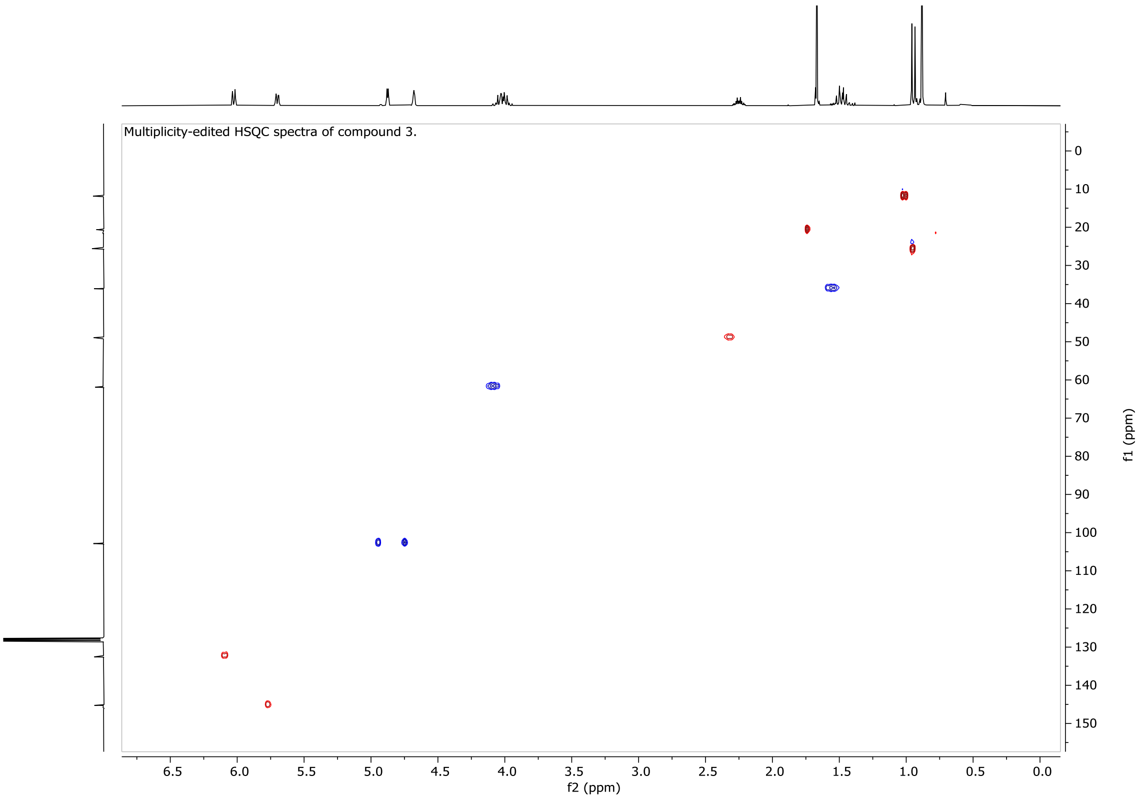

Figure S28. ORTEP diagram for compound **15**.

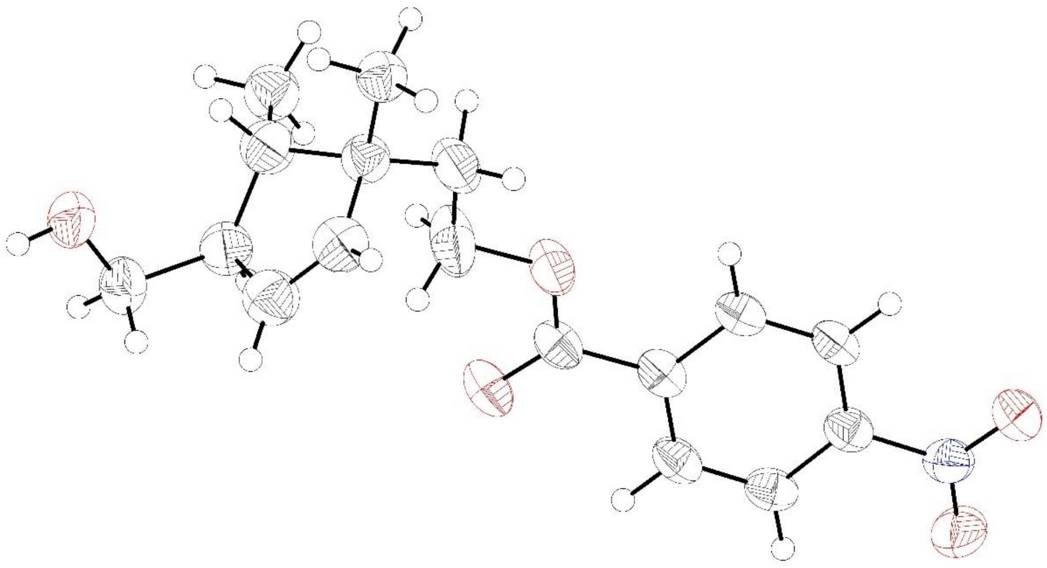

Thermal ellipsoids are shown with 50% probability. For details, see CCDC 2327985 (Cambridge Crystallographic Data Centre).

Figure S29.

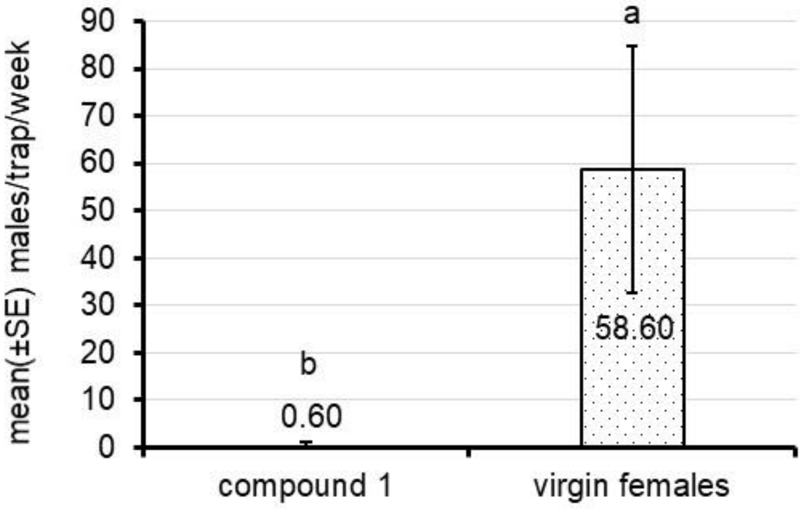

Methods. The response of *P. longispinus* males to 2-(1,5,5-trimethylcyclopent-2-en-1-yl)ethyl acetate **1** (the pheromone compound described by Millar et al.[20]) and conspecific virgin females was evaluated in a preliminary field trial carried out in a persimmon orchard, located in the municipality of L'Alcúdia (Valencia, Spain). Racemic compound **1** was emitted employing rubber septa (Ecología y Protección Agrícola SL, Carlet, Spain), which were loaded with 100 µg by impregnation with the corresponding hexane solution. Both rubber septa and females were installed in Delta traps, provided with a sticky base (Biagro SL, Massalfassar, Spain). Groups of 15-25 virgin females from the laboratory colony were placed inside muslin bags, which were hung in the center of the Delta traps to be employed as baits. Three blocks were installed and traps were hung at a height of 1.5 m and were spaced 20 m apart, with each block at least 30 m apart. The traps were revised weekly and the number of captured males was counted under a stereomicroscope (Stemi 508; Zeiss, Oberkochen, Germany) at 50X magnification.

Table data and structure refinement for compound 15

Identification code	b20230196_COS1remDUCu
Empirical formula	$C_{17}H_{21}NO_5$
Formula weight	319.35
Temperature/K	169.99(10)
Crystal system	triclinic
Space group	P-1
a/Å	7.4355(3)
b/Å	12.4522(4)
c/Å	18.1636(9)
α/°	98.606(4)
β/°	91.155(4)
γ/°	90.987(3)
Volume/Å^3	1662.12(12)
Z	4
ρ_{calc}g/cm^3	1.276
μ/mm^{-1}	0.779
F(000)	680.0
Crystal size/mm^3	0.396 × 0.214 × 0.083
Radiation	CuKα (λ = 1.54184)
2Θ range for data collection/°	7.182 to 147.446
Index ranges	$-9 \le h \le 9$, $-15 \le k \le 9$, $-22 \le l \le 22$
Reflections collected	15069
Independent reflections	6480 [R_{int} = 0.0443, R_{sigma} = 0.0693]
Data/restraints/parameters	6480/214/559
Goodness-of-fit on F^2	1.082

Final R indexes [I>=2σ (I)]	R_1 = 0.0964, wR_2 = 0.2407
Final R indexes [all data]	R_1 = 0.1400, wR_2 = 0.2690
Largest diff. peak/hole / e Å^{-3}	0.34/-0.25

[a] Esquema de pesado: $1/[\sigma^2(F_o^2)+(0.1091P)^2 + 1.1836P]$ donde $P = [Max(F_o^2,0)+2F_c^2]/3$.
[b] Expresión de extinción secundaria tipo SHELXL: $F_c^* = kF_c[1+0.001F_c^2\lambda^3/sen(2\theta)]^{-1/4}$

Table 2 Fractional Atomic Coordinates (×10^4) and Equivalent Isotropic Displacement Parameters (Å^2×10^3) for b20230196_COS1remDUCu. U_{eq} is defined as 1/3 of of the trace of the orthogonalised U_{IJ} tensor.

Atom	*x*	*y*	*z*	U(eq)
O2A	7005(4)	5675(2)	8280.1(16)	55.0(7)
O3A	5674(4)	6946(2)	9078.6(17)	55.1(7)
O4A	9444(4)	2914(2)	11123.3(17)	59.4(8)
O5A	8165(5)	4110(3)	11909.0(17)	67.9(9)
N1A	8580(4)	3745(3)	11278.6(19)	48.7(8)
C1A	6029(6)	8287(3)	6732(3)	55.7(10)
C9A	7481(7)	5841(3)	7001(3)	61.0(12)
C10A	6574(8)	6313(4)	7692(3)	73.4(15)
C11A	6511(5)	6113(3)	8954(2)	43.3(8)
C12A	7083(5)	5473(3)	9549(2)	41.9(8)
C13A	7815(5)	4442(3)	9378(2)	42.1(8)
C14A	8325(5)	3872(3)	9946(2)	41.7(8)
C15A	8066(5)	4343(3)	10670(2)	42.3(8)
C16A	7322(5)	5360(3)	10855(2)	44.3(8)
C17A	6840(5)	5926(3)	10283(2)	44.1(9)
O1A	5680(40)	9400(40)	5770(12)	65(4)
C2A	7693(10)	7681(6)	6476(5)	56(2)
C3A	7197(11)	6463(7)	6310(5)	52(2)
C4A	5237(13)	6497(8)	6092(6)	84(3)
C5A	4566(12)	7513(6)	6339(5)	79(3)
C6A	5100(30)	9207(12)	6486(7)	60(4)
C7A	9283(14)	7969(9)	7014(7)	82(3)
C8A	8034(17)	5834(9)	5654(7)	86(3)
O1C	6170(30)	9450(30)	5736(10)	65(4)
C2C	6004(15)	7202(8)	6234(6)	58(3)
C3C	7797(17)	6618(9)	6438(7)	48(3)
C4C	8982(17)	7569(9)	6671(7)	61(3)
C5C	8051(15)	8455(9)	6884(7)	63(3)
C6C	5940(30)	9436(8)	6491(5)	59(3)
C7C	4269(17)	6602(9)	6229(7)	63(3)
C8C	8776(17)	5999(9)	5744(7)	51(3)
O2B	-2055(4)	9415(2)	1847.4(15)	49.0(7)
O3B	-510(4)	8167(2)	1104.3(17)	54.2(7)
O4B	-3183(4)	10656(2)	-1878.7(17)	61.8(8)
O5B	-4239(4)	11982(2)	-1122.9(17)	56.8(8)

N1B	-3510(4)	11100(2)	-1250.4(19)	45.8(7)
C1B	934(6)	8136(4)	3876(3)	58.9(11)
C3B	-1823(6)	7349(4)	3236(3)	60.5(11)
C8B	-3064(7)	6364(4)	3248(3)	66.4(12)
C9B	-2449(5)	7937(3)	2592(2)	50.3(9)
C10B	-1398(6)	8952(3)	2492(2)	54.8(10)
C11B	-1461(5)	8951(3)	1191(2)	43.7(9)
C12B	-2079(5)	9522(3)	563(2)	40.9(8)
C13B	-1803(5)	9020(3)	-153(2)	44.1(9)
C14B	-2267(5)	9530(3)	-755(2)	45.7(9)
C15B	-3022(5)	10550(3)	-616(2)	38.7(8)
C16B	-3322(5)	11066(3)	94(2)	41.1(8)
C17B	-2846(5)	10548(3)	688(2)	40.7(8)
O1B	3690(20)	8594(12)	4577(9)	69.0(17)
C2B	77(8)	7162(5)	3372(4)	50.9(16)
C4B	-2221(10)	8199(6)	3969(4)	62.4(19)
C5B	-756(10)	8599(6)	4292(4)	62.0(19)
C6B	2190(40)	7820(20)	4551(17)	80(3)
C7B	1046(9)	6811(5)	2666(4)	53.3(17)
O1D	3074(11)	8792(6)	4865(4)	69.0(17)
C0AA	-2150(30)	8740(19)	4268(14)	99(7)
C2D	-900(20)	7800(12)	3911(8)	60(4)
C4D	-29(13)	6838(10)	2863(7)	54(4)
C5D	1499(16)	7295(9)	3205(6)	57(4)
C6D	2419(19)	7929(10)	4376(7)	80(3)

Table 3 Anisotropic Displacement Parameters (Å^2×10^3) for b20230196_COS1remDUCu. The Anisotropic displacement factor exponent takes the form: $-2\pi^2[h^2a^{*2}U_{11}+2hka^*b^*U_{12}+\ldots]$.

Atom	U_{11}	U_{22}	U_{33}	U_{23}	U_{13}	U_{12}
O2A	72.2(19)	41.8(13)	54.1(17)	13.9(12)	14.6(14)	14.0(13)
O3A	58.6(17)	37.4(13)	70(2)	7.9(12)	14.5(14)	11.5(12)
O4A	70.0(19)	46.8(15)	60.1(19)	2.7(13)	-1.4(15)	15.5(14)
O5A	88(2)	71.5(19)	41.5(17)	-2.1(14)	-1.0(16)	21.5(17)
N1A	52.3(19)	46.3(17)	44.6(19)	-2.3(14)	-2.5(15)	3.6(15)
C1A	56.6(13)	55.1(13)	55.6(13)	8.8(9)	1.2(9)	2.5(9)
C9A	82(3)	42(2)	60(3)	10.6(19)	9(2)	13(2)
C10A	98(4)	62(3)	68(3)	30(2)	27(3)	34(3)
C11A	45(2)	34.5(17)	50(2)	4.8(15)	10.8(17)	-1.2(15)
C12A	30.3(17)	36.6(17)	58(2)	4.7(16)	7.7(16)	0.3(14)
C13A	40.8(19)	36.9(17)	47(2)	-0.7(15)	8.6(16)	-1.3(15)
C14A	37.3(18)	30.3(15)	56(2)	0.9(15)	4.5(16)	0.8(14)
C15A	36.1(19)	40.1(17)	49(2)	1.6(15)	2.2(16)	-1.4(15)
C16A	41(2)	43.0(18)	46(2)	-4.4(16)	6.1(16)	0.9(15)

C17A	36.2(19)	34.5(16)	59(2)	-1.8(16)	5.6(17)	2.9(14)
O1A	80(12)	50(3)	66(2)	20.8(19)	-17(4)	-7(8)
C2A	56(2)	56(2)	56(2)	9.0(10)	1.6(10)	1.5(10)
C3A	53(2)	51(2)	52(2)	7.4(10)	1.1(10)	1.6(10)
C4A	85(3)	84(3)	84(3)	13.2(11)	1.8(10)	1.0(10)
C5A	79(3)	79(3)	79(3)	13.3(11)	1.4(10)	1.3(10)
C6A	60(4)	60(4)	60(4)	9.1(12)	1.4(10)	1.6(10)
C7A	81(3)	82(3)	83(3)	13(2)	2(2)	1.6(19)
C8A	88(4)	86(4)	85(4)	14(2)	2(2)	3(2)
O1C	80(12)	50(3)	66(2)	20.8(19)	-17(4)	-7(8)
C2C	58(3)	58(3)	57(3)	8.6(11)	1.6(10)	1.1(10)
C3C	48(3)	48(3)	48(3)	7.1(11)	1.1(10)	1.4(10)
C4C	61(3)	62(3)	61(3)	9.3(11)	1.1(10)	1.4(10)
C5C	63(3)	63(3)	63(3)	9.7(11)	1.3(10)	1.1(10)
C6C	71(7)	50(4)	59(5)	18(4)	0(4)	6(4)
C7C	63(4)	63(4)	63(4)	9(2)	2(2)	3(2)
C8C	52(3)	52(3)	51(3)	8.1(19)	2.8(19)	2.2(19)
O2B	56.9(16)	40.4(13)	49.5(16)	5.1(11)	0.6(13)	9.1(12)
O3B	46.1(15)	45.9(14)	73(2)	15.6(13)	9.1(14)	14.2(12)
O4B	74(2)	64.7(17)	44.8(17)	0.1(14)	-3.9(15)	9.2(15)
O5B	64.6(18)	43.6(14)	63.2(19)	10.5(13)	-2.1(15)	11.8(13)
N1B	42.2(17)	45.3(16)	48.3(19)	2.2(14)	-2.2(14)	-1.4(14)
C1B	60.0(14)	57.2(13)	59.8(14)	10.1(9)	-0.4(9)	0.6(9)
C3B	61.6(14)	59.4(13)	61.0(14)	10.7(9)	-0.1(9)	-1.4(9)
C8B	66(3)	63(3)	71(3)	15(2)	7(2)	-16(2)
C9B	44(2)	48(2)	58(3)	5.0(18)	5.0(18)	-1.7(17)
C10B	69(3)	42.7(19)	53(2)	7.3(17)	-9(2)	3.4(19)
C11B	37.7(19)	36.1(17)	57(2)	7.9(16)	-2.4(17)	-2.5(15)
C12B	29.1(17)	39.2(17)	53(2)	3.2(16)	2.7(16)	-0.9(14)
C13B	35.4(18)	33.9(16)	62(2)	4.0(16)	0.7(17)	4.5(14)
C14B	38.7(19)	42.6(18)	52(2)	-5.1(16)	6.0(17)	3.5(15)
C15B	34.9(18)	38.9(17)	41.4(19)	3.8(14)	-3.2(15)	-4.5(14)
C16B	37.8(18)	32.0(16)	52(2)	2.2(15)	0.9(16)	2.2(14)
C17B	37.0(18)	36.6(17)	47(2)	2.8(15)	4.1(16)	0.5(14)
O1B	88(5)	71(3)	46(4)	6(3)	-9(3)	-13(3)
C2B	51.8(19)	50.3(18)	50.7(18)	7.6(10)	1.3(10)	1.0(10)
C4B	62(2)	62(2)	63(2)	8.8(10)	1.8(10)	1.2(10)
C5B	62(2)	61(2)	62(2)	7.9(10)	1.6(10)	1.2(10)
C6B	88(4)	76(4)	82(6)	39(4)	-19(4)	-19(3)
C7B	57(2)	48(2)	55(2)	8.2(16)	3.3(17)	7.5(17)
O1D	88(5)	71(3)	46(4)	6(3)	-9(3)	-13(3)
C0AA	99(7)	98(7)	99(7)	13(2)	2(2)	1(2)
C2D	61(4)	60(4)	60(4)	9.0(12)	1.5(10)	1.4(10)
C4D	54(4)	54(4)	54(4)	8.6(12)	1.0(10)	0.9(10)
C5D	57(4)	57(4)	57(4)	9.3(12)	0.8(10)	1.3(10)

C6D	88(4)	76(4)	82(6)	39(4)	-19(4)	-19(3)

Table 4 Bond Lengths for b20230196_COS1remDUCu.

Atom	Atom	Length/Å	Atom	Atom	Length/Å
O2A	C10A	1.457(5)	C4C	C5C	1.325(17)
O2A	C11A	1.325(5)	O2B	C10B	1.460(5)
O3A	C11A	1.213(4)	O2B	C11B	1.332(5)
O4A	N1A	1.227(4)	O3B	C11B	1.208(4)
O5A	N1A	1.215(4)	O4B	N1B	1.222(4)
N1A	C15A	1.470(5)	O5B	N1B	1.225(4)
C1A	C2A	1.505(8)	N1B	C15B	1.469(5)
C1A	C5A	1.532(8)	C1B	C2B	1.527(8)
C1A	C6A	1.469(12)	C1B	C5B	1.554(8)
C1A	C2C	1.509(11)	C1B	C6B	1.62(3)
C1A	C5C	1.529(12)	C1B	C2D	1.426(15)
C1A	C6C	1.559(10)	C1B	C5D	1.553(9)
C9A	C10A	1.485(6)	C1B	C6D	1.466(14)
C9A	C3A	1.582(9)	C3B	C8B	1.525(6)
C9A	C3C	1.527(11)	C3B	C9B	1.538(6)
C11A	C12A	1.496(5)	C3B	C2B	1.458(7)
C12A	C13A	1.397(5)	C3B	C4B	1.609(9)
C12A	C17A	1.384(5)	C3B	C2D	1.426(16)
C13A	C14A	1.388(5)	C3B	C4D	1.606(9)
C14A	C15A	1.377(5)	C9B	C10B	1.510(5)
C15A	C16A	1.388(5)	C11B	C12B	1.501(5)
C16A	C17A	1.385(5)	C12B	C13B	1.377(5)
O1A	C6A	1.432(10)	C12B	C17B	1.397(5)
C2A	C3A	1.539(11)	C13B	C14B	1.385(5)
C2A	C7A	1.521(14)	C14B	C15B	1.388(5)
C3A	C4A	1.505(9)	C15B	C16B	1.375(5)
C3A	C8A	1.479(14)	C16B	C17B	1.381(5)
C4A	C5A	1.382(9)	O1B	C6B	1.450(10)
O1C	C6C	1.387(17)	C2B	C7B	1.496(9)
C2C	C3C	1.594(15)	C4B	C5B	1.283(11)
C2C	C7C	1.479(16)	O1D	C6D	1.364(13)
C3C	C4C	1.469(17)	C0AA	C2D	1.58(3)
C3C	C8C	1.574(16)	C4D	C5D	1.358(9)

Table 5 Bond Angles for b20230196_COS1remDUCu.

Atom	Atom	Atom	Angle/°	Atom	Atom	Atom	Angle/°
C11A	O2A	C10A	114.6(3)	O1C	C6C	C1A	114.7(17)
O4A	N1A	C15A	118.0(3)	C11B	O2B	C10B	115.6(3)

O5A	N1A	O4A	123.5(3)	O4B	N1B	O5B	123.2(3)
O5A	N1A	C15A	118.5(3)	O4B	N1B	C15B	118.7(3)
C2A	C1A	C5A	100.6(5)	O5B	N1B	C15B	118.2(3)
C6A	C1A	C2A	133.2(8)	C2B	C1B	C5B	99.9(4)
C6A	C1A	C5A	89.6(9)	C2B	C1B	C6B	114.5(9)
C2C	C1A	C5C	100.3(7)	C5B	C1B	C6B	102.2(12)
C2C	C1A	C6C	127.6(6)	C2D	C1B	C5D	98.7(8)
C5C	C1A	C6C	89.7(9)	C2D	C1B	C6D	126.9(8)
C10A	C9A	C3A	115.5(4)	C6D	C1B	C5D	96.0(8)
C10A	C9A	C3C	115.5(5)	C8B	C3B	C9B	108.1(4)
O2A	C10A	C9A	109.1(3)	C8B	C3B	C4B	107.1(4)
O2A	C11A	C12A	113.1(3)	C8B	C3B	C4D	103.8(5)
O3A	C11A	O2A	123.8(4)	C9B	C3B	C4B	103.7(4)
O3A	C11A	C12A	123.1(4)	C9B	C3B	C4D	97.6(6)
C13A	C12A	C11A	121.7(3)	C2B	C3B	C8B	114.9(4)
C17A	C12A	C11A	117.8(3)	C2B	C3B	C9B	121.5(4)
C17A	C12A	C13A	120.4(3)	C2B	C3B	C4B	99.6(5)
C14A	C13A	C12A	120.0(4)	C2D	C3B	C8B	118.1(7)
C15A	C14A	C13A	118.3(3)	C2D	C3B	C9B	128.1(7)
C14A	C15A	N1A	119.0(3)	C2D	C3B	C4D	93.2(8)
C14A	C15A	C16A	122.9(4)	C10B	C9B	C3B	116.4(4)
C16A	C15A	N1A	118.1(3)	O2B	C10B	C9B	111.7(3)
C17A	C16A	C15A	118.2(4)	O2B	C11B	C12B	112.5(3)
C12A	C17A	C16A	120.2(3)	O3B	C11B	O2B	124.4(4)
C1A	C2A	C3A	108.1(6)	O3B	C11B	C12B	123.1(4)
C1A	C2A	C7A	112.4(7)	C13B	C12B	C11B	117.7(3)
C7A	C2A	C3A	115.0(7)	C13B	C12B	C17B	120.2(3)
C2A	C3A	C9A	113.7(7)	C17B	C12B	C11B	122.0(3)
C4A	C3A	C9A	111.5(7)	C12B	C13B	C14B	120.4(3)
C4A	C3A	C2A	101.3(7)	C13B	C14B	C15B	118.2(4)
C8A	C3A	C9A	108.6(7)	C14B	C15B	N1B	118.7(3)
C8A	C3A	C2A	116.6(8)	C16B	C15B	N1B	118.8(3)
C8A	C3A	C4A	104.5(9)	C16B	C15B	C14B	122.5(3)
C5A	C4A	C3A	110.2(8)	C15B	C16B	C17B	118.6(3)
C4A	C5A	C1A	111.5(8)	C16B	C17B	C12B	120.1(4)
O1A	C6A	C1A	111.6(19)	C3B	C2B	C1B	110.4(4)
C1A	C2C	C3C	105.8(8)	C3B	C2B	C7B	112.0(5)
C7C	C2C	C1A	113.4(9)	C7B	C2B	C1B	114.8(5)
C7C	C2C	C3C	118.6(9)	C5B	C4B	C3B	111.3(7)
C9A	C3C	C2C	112.5(9)	C4B	C5B	C1B	112.0(7)
C9A	C3C	C8C	109.2(8)	O1B	C6B	C1B	102.6(13)
C4C	C3C	C9A	117.5(10)	C1B	C2D	C3B	118.4(11)
C4C	C3C	C2C	100.2(9)	C1B	C2D	C0AA	113.4(13)
C4C	C3C	C8C	103.0(10)	C3B	C2D	C0AA	104.3(13)
C8C	C3C	C2C	114.1(10)	C5D	C4D	C3B	112.9(9)

C5C	C4C	C3C	111.7(11)	C4D	C5D	C1B	107.5(9)
C4C	C5C	C1A	113.1(10)	O1D	C6D	C1B	117.1(10)

Table 6 Hydrogen Atom Coordinates (Å×10[4]) and Isotropic Displacement Parameters (Å[2]×10[3]) for b20230196_COS1remDUCu.

Atom	*x*	*y*	*z*	U(eq)
H1A	5930.51	8327.22	7283.25	67
H1AA	5360.73	8253.13	7200.19	67
H9AA	7042.84	5081.52	6858.11	73
H9AB	8788.45	5819.17	7111.16	73
H9AC	6747.71	5213.16	6753.83	73
H9AD	8658.83	5561.47	7140.7	73
H10A	6986.67	7075.97	7844.17	88
H10B	5256.05	6306.61	7602.38	88
H13A	7963.58	4131.49	8873.18	50
H14A	8840.01	3174.49	9838.1	50
H16A	7149.25	5660.62	11360.43	53
H17A	6339.54	6626.87	10393.82	53
H1AB	5151.33	8960.48	5436.69	97
H2A	8028.46	7914.3	5992.96	67
H4A	4560.19	5906.79	5823.61	101
H5A	3348.9	7706.86	6273.57	95
H6AA	3783.82	9054.7	6462.36	72
H6AB	5330.23	9867.41	6854.43	72
H7AA	9743.74	8697.51	6968.77	123
H7AB	10234.44	7440.09	6893.53	123
H7AC	8895.62	7953.11	7524.43	123
H8AA	9200.63	5569.48	5802.46	129
H8AB	8209.09	6301.55	5271.8	129
H8AC	7247.51	5215.26	5454.21	129
H1C	6191.65	10095.64	5652.8	97
H6CA	4870(60)	9870(50)	6630(50)	77
H2C	6153.34	7364.37	5715.31	69
H4C	10258.62	7551.05	6669.48	74
H5C	8586.18	9126.29	7108.23	75
H6CB	7030(60)	9830(50)	6720(40)	71
H7CA	3341.72	6978.49	5980.37	95
H7CB	3921.35	6565.66	6742.15	95
H7CC	4393.95	5864.54	5960.52	95
H8CA	10049.14	5920.6	5872.47	77
H8CB	8675.6	6413.13	5327.62	77
H8CC	8214.19	5278.49	5601.17	77
H1B	1531.26	8665.36	3591.53	71
H1BA	1024.59	8891.12	3753.45	71

H8BA	-4317.29	6595.81	3264.42	100
H8BB	-2778.4	6026.53	3688.53	100
H8BC	-2893.42	5838.16	2797.63	100
H9BA	-2400.44	7419.51	2122.98	60
H9BB	-3723.64	8131.93	2669.03	60
H10C	-1496.37	9496.83	2945.56	66
H10D	-112.18	8775.73	2429.15	66
H13B	-1292.25	8319.33	-234.49	53
H14B	-2072.58	9191.08	-1250.68	55
H16B	-3844.75	11762.84	172.98	49
H17B	-3040.86	10890.14	1182.68	49
H6DA	1790(40)	7310(20)	4671(15)	49
H1D	2860(80)	8960(40)	5340(11)	61
H1BB	3295.47	9205.65	4519.61	103
H2B	122.54	6538.3	3660.82	61
H4B	-3390.55	8381.42	4141.51	75
H5B	-709.86	9117.61	4732.63	74
H6BA	1400(40)	8020(50)	4956(13)	96
H6BB	2500(60)	7105(16)	4350(30)	700(700)
H7BA	450.04	6159.13	2391.71	80
H7BB	2294.71	6648.71	2783.35	80
H7BC	1026.67	7395.01	2359.27	80
H0AA	-1892.18	9400.35	4053.49	148
H0AB	-1930.29	8882.11	4808	148
H0AC	-3416.96	8520.16	4165.34	148
H2D	-930.87	7230.16	4244.53	73
H4D	-35.69	6275.97	2447.14	65
H5D	2695.68	7131.04	3059.67	68
H6DB	3400(80)	7640(40)	4090(40)	96

Table 7 Atomic Occupancy for b20230196_COS1remDUCu.

**Atom**	***Occupancy***	**Atom**	***Occupancy***	**Atom**	***Occupancy***
H1A	0.580(8)	H1AA	0.420(8)	H9AA	0.580(8)
H9AB	0.580(8)	H9AC	0.420(8)	H9AD	0.420(8)
O1A	0.46(3)	H1AB	0.46(3)	C2A	0.580(8)
H2A	0.580(8)	C3A	0.580(8)	C4A	0.580(8)
H4A	0.580(8)	C5A	0.580(8)	H5A	0.580(8)
C6A	0.46(3)	H6AA	0.46(3)	H6AB	0.46(3)
C7A	0.580(8)	H7AA	0.580(8)	H7AB	0.580(8)
H7AC	0.580(8)	C8A	0.580(8)	H8AA	0.580(8)
H8AB	0.580(8)	H8AC	0.580(8)	O1C	0.54(3)
H1C	0.54(3)	H6CA	0.54(3)	C2C	0.420(8)
H2C	0.420(8)	C3C	0.420(8)	C4C	0.420(8)

H4C	0.420(8)	C5C	0.420(8)	H5C	0.420(8)
C6C	0.54(3)	H6CB	0.54(3)	C7C	0.420(8)
H7CA	0.420(8)	H7CB	0.420(8)	H7CC	0.420(8)
C8C	0.420(8)	H8CA	0.420(8)	H8CB	0.420(8)
H8CC	0.420(8)	H1B	0.682(8)	H1BA	0.318(8)
H6DA	0.671(12)	H1D	0.671(12)	O1B	0.329(12)
H1BB	0.329(12)	C2B	0.682(8)	H2B	0.682(8)
C4B	0.682(8)	H4B	0.682(8)	C5B	0.682(8)
H5B	0.682(8)	C6B	0.329(12)	H6BA	0.329(12)
H6BB	0.329(12)	C7B	0.682(8)	H7BA	0.682(8)
H7BB	0.682(8)	H7BC	0.682(8)	O1D	0.671(12)
C0AA	0.318(8)	H0AA	0.318(8)	H0AB	0.318(8)
H0AC	0.318(8)	C2D	0.318(8)	H2D	0.318(8)
C4D	0.318(8)	H4D	0.318(8)	C5D	0.318(8)
H5D	0.318(8)	C6D	0.671(12)	H6DB	0.671(12)

Experimental

Single crystals of $C_{17}H_{21}NO_5$ **[b20230196_COS1remDUCu]**. A suitable crystal was selected and mounted on a **SuperNova, Dual, Cu at home/near, HyPix** diffractometer. The crystal was kept at 169.99(10) K during data collection. Using Olex2 [1], the structure was solved with the ShelXT [2] structure solution program using Intrinsic Phasing and refined with the ShelXL [3] refinement package using Least Squares minimisation.

1. Dolomanov, O.V., Bourhis, L.J., Gildea, R.J, Howard, J.A.K. & Puschmann, H. (2009), J. Appl. Cryst. 42, 339-341.
2. Sheldrick, G.M. (2015). Acta Cryst. A71, 3-8.
3. Sheldrick, G.M. (2015). Acta Cryst. C71, 3-8.

Crystal structure determination of [b20230196_COS1remDUCu]

Crystal Data for $C_{17}H_{21}NO_5$ (M =319.35 g/mol): triclinic, space group P-1 (no. 2), a = 7.4355(3) Å, b = 12.4522(4) Å, c = 18.1636(9) Å, α = 98.606(4)°, β = 91.155(4)°, γ = 90.987(3)°, V = 1662.12(12) Å^3, Z = 4, T = 169.99(10) K, μ(CuKα) = 0.779 mm^{-1}, $Dcalc$ = 1.276 g/cm^3, 15069 reflections measured (7.182° ≤ 2Θ ≤ 147.446°), 6480 unique (R_{int} = 0.0443, R_{sigma} = 0.0693) which were used in all calculations. The final R_1 was 0.0964 (I > 2σ(I)) and wR_2 was 0.2690 (all data).

Refinement model description

Number of restraints - 214, number of constraints - unknown.

Details:

```
1. Fixed Uiso
 At 1.2 times of:
  All C(H,H) groups, H13A of C13A, H14A of C14A, H16A of C16A, H17A of C17A,
  H6DB of C6D, H5D of C5D, H4D of C4D, H2D of C2D, H13B of C13B, H14B of C14B,
  H16B of C16B, H2B of C2B, H4B of C4B, H5B of C5B, H6BA of C6B, H2A of C2A,
  H4A of C4A, H5A of C5A, H6CA of O1C, H2C of C2C, H4C of C4C, H5C of C5C,
H6CB
  of C6C, All C(H,H,H,H) groups
 At 1.5 times of:
  All C(H,H,H) groups, H1D of C17B, H1BB of O1B, H1AB of O1A, H1C of O1C
2. Restrained distances
 C1A-C5A
 1.54 with sigma of 0.01
 C5A-C4A
 1.4 with sigma of 0.01
 C4A-C3A
 1.54 with sigma of 0.01
 C4D-C3B
 1.54 with sigma of 0.01
```

C5D-C1B
1.54 with sigma of 0.01
C5D-C4D
1.35 with sigma of 0.01
H6CA-C6C
0.99 with sigma of 0.01
H6CB-C6C
0.99 with sigma of 0.01
H1BB-O1B
0.85 with sigma of 0.01
O1A-C6A
1.43 with sigma of 0.01
H1D-O1D
0.88 with sigma of 0.01
H6DB-C6D
0.95 with sigma of 0.01
H6BA-C6B
0.95 with sigma of 0.01
O1D-C6B
1.39 with sigma of 0.01
O1B-C6B
1.43 with sigma of 0.01
H6BB-C6B
0.95 with sigma of 0.01
H6DA-C6D
0.95 with sigma of 0.01
H6CB-H6CA
1.62 with sigma of 0.02
H6BA-C1B
2.02 with sigma of 0.02
H6BA-O1B
2.02 with sigma of 0.02
H1BB-C6B
2.02 with sigma of 0.02
H6BB-C1B
2.02 with sigma of 0.02
H6BA-H6BB
1.69 with sigma of 0.02
H6BB-O1B
2.02 with sigma of 0.02
H6DB-H6DA
1.69 with sigma of 0.02
H6DA-C6D
1.8 with sigma of 0.02
H6DA-C1B
1.95 with sigma of 0.02
H1D-C6D
2.02 with sigma of 0.02
H6DA-O1D
2.02 with sigma of 0.02

3. Uiso/Uaniso restraints and constraints

Uanis(C7B) ≈ Ueq, Uanis(C2B) ≈ Ueq, Uanis(C3B) ≈ Ueq, Uanis(C4B) ≈ Ueq, Uanis(C5B) ≈ Ueq, Uanis(C1B) ≈ Ueq: with sigma of 0.001 and sigma for terminal atoms of 0.002
Uanis(C7A) ≈ Ueq: with sigma of 0.001 and sigma for terminal atoms of 0.002
Uanis(C2C) ≈ Ueq, Uanis(C5C) ≈ Ueq, Uanis(C3C) ≈ Ueq, Uanis(C8A) ≈ Ueq, Uanis(C7C) ≈ Ueq, Uanis(C4C) ≈ Ueq: with sigma of 0.001 and sigma for terminal atoms of 0.002
Uanis(C2D) ≈ Ueq, Uanis(C0AA) ≈ Ueq, Uanis(C4D) ≈ Ueq, Uanis(C5D) ≈ Ueq: with sigma of 0.001 and sigma for terminal atoms of 0.002
Uanis(C5A) ≈ Ueq, Uanis(C4A) ≈ Ueq, Uanis(C3A) ≈ Ueq, Uanis(C2A) ≈ Ueq, Uanis(C1A) ≈ Ueq, Uanis(C6A) ≈ Ueq, Uanis(C8C) ≈ Ueq: with sigma of 0.001 and sigma for terminal atoms of 0.002
Uanis(C6A) ≈ Ueq, Uanis(O1A) ≈ Ueq, Uanis(C6C) ≈ Ueq, Uanis(O1C) ≈ Ueq: with sigma of 0.01 and sigma for terminal atoms of 0.02
Uanis(O1D) ≈ Ueq, Uanis(C6B) ≈ Ueq: with sigma of 0.01 and sigma for terminal atoms of 0.02
Uanis(C6D) ≈ Ueq, Uanis(O1D) ≈ Ueq, Uanis(O1B) ≈ Ueq: with sigma of

```
0.01 and sigma for terminal atoms of 0.02
Uanis(O1A) = Uanis(O1C)
Uanis(O1D) = Uanis(O1B)
Uanis(C6D) = Uanis(C6B)
4. Others
 Sof(H1AA)=Sof(H9AC)=Sof(H9AD)=Sof(C2C)=Sof(H2C)=Sof(C3C)=Sof(C4C)=Sof(H4C)=
 Sof(C5C)=Sof(H5C)=Sof(C7C)=Sof(H7CA)=Sof(H7CB)=Sof(H7CC)=Sof(C8C)=Sof(H8CA)=
 Sof(H8CB)=Sof(H8CC)=1-FVAR(1)
 Sof(H1A)=Sof(H9AA)=Sof(H9AB)=Sof(C2A)=Sof(H2A)=Sof(C3A)=Sof(C4A)=Sof(H4A)=
 Sof(C5A)=Sof(H5A)=Sof(C7A)=Sof(H7AA)=Sof(H7AB)=Sof(H7AC)=Sof(C8A)=Sof(H8AA)=
 Sof(H8AB)=Sof(H8AC)=FVAR(1)
 Sof(H1BA)=Sof(C0AA)=Sof(H0AA)=Sof(H0AB)=Sof(H0AC)=Sof(C2D)=Sof(H2D)=Sof(C4D)=
 Sof(H4D)=Sof(C5D)=Sof(H5D)=1-FVAR(2)
 Sof(H1B)=Sof(C2B)=Sof(H2B)=Sof(C4B)=Sof(H4B)=Sof(C5B)=Sof(H5B)=Sof(C7B)=
 Sof(H7BA)=Sof(H7BB)=Sof(H7BC)=FVAR(2)
 Sof(O1C)=Sof(H1C)=Sof(H6CA)=Sof(C6C)=Sof(H6CB)=1-FVAR(3)
 Sof(O1A)=Sof(H1AB)=Sof(C6A)=Sof(H6AA)=Sof(H6AB)=FVAR(3)
 Sof(H6DA)=Sof(H1D)=Sof(O1D)=Sof(C6D)=Sof(H6DB)=1-FVAR(4)
 Sof(O1B)=Sof(H1BB)=Sof(C6B)=Sof(H6BA)=Sof(H6BB)=FVAR(4)
5.a Ternary CH refined with riding coordinates:
 C1A(H1A), C1A(H1AA), C2A(H2A), C2C(H2C), C1B(H1B), C1B(H1BA), C2B(H2B),
 C2D(H2D)
5.b Secondary CH2 refined with riding coordinates:
 C9A(H9AA,H9AB), C9A(H9AC,H9AD), C10A(H10A,H10B), C6A(H6AA,H6AB), C9B(H9BA,
 H9BB), C10B(H10C,H10D)
5.c Aromatic/amide H refined with riding coordinates:
 C13A(H13A), C14A(H14A), C16A(H16A), C17A(H17A), C4A(H4A), C5A(H5A), C4C(H4C),
 C5C(H5C), C13B(H13B), C14B(H14B), C16B(H16B), C17B(H17B), C4B(H4B), C5B(H5B),
 C4D(H4D), C5D(H5D)
5.d Idealised Me refined as rotating group:
 C7A(H7AA,H7AB,H7AC), C8A(H8AA,H8AB,H8AC), C7C(H7CA,H7CB,H7CC), C8C(H8CA,H8CB,
 H8CC), C8B(H8BA,H8BB,H8BC), C7B(H7BA,H7BB,H7BC), C0AA(H0AA,H0AB,H0AC)
5.e Idealised tetrahedral OH refined as rotating group:
 O1A(H1AB), O1C(H1C), O1B(H1BB)
```

This report has been created with Olex2, compiled on 2018.05.29 svn.r3508 for OlexSys. Please let us know if there are any errors or if you would like to have additional features.
